# A multi-omics approach to elucidate the mechanisms of action of a dietary muramidase administered to broiler chickens

**DOI:** 10.1038/s41598-022-09546-6

**Published:** 2022-04-01

**Authors:** Giorgio Brugaletta, Alessandra De Cesare, Luca Laghi, Gerardo Manfreda, Marco Zampiga, Chiara Oliveri, Estefanía Pérez-Calvo, Gilberto Litta, Susanna Lolli, Federico Sirri

**Affiliations:** 1 Department of Agricultural and Food Sciences, Alma Mater Studiorum – University of Bologna, Ozzano dell’Emilia, Bologna, 40064 Italy; 2 Department of Veterinary Medical Sciences, Alma Mater Studiorum – University of Bologna, Ozzano dell’Emilia, Bologna, 40064 Italy; 3 Research Center for Animal Nutrition and Health, DSM Nutritional Products, Village-Neuf, Saint Louis, 68305 France; 4 DSM Nutritional Products, Animal Nutrition and Health, Segrate, Milano, 20054 Italy

**Keywords:** DNA, Enzymes, Metabolomics, Microbiology, Molecular biology, Systems biology, Animal physiology

## Abstract

A novel dietary muramidase has been shown to have positive effects on broiler chickens. However, very little is known about its mechanisms of action. The present multi-omics investigation sought to address this knowledge gap. A total of 2,340 day-old male broilers were assigned to 3 groups (12 replicates each) fed, from 0 to 42 d, a basal diet (control group—CON) or the basal diet supplemented with muramidase at 25,000 (low-dose group—MUL) or 45,000 LSU(F)/kg feed (high-dose group—MUH). MUH significantly outperformed CON in terms of cumulative feed intake (4,798 *vs* 4,705 g), body weight (2,906 *vs* 2,775 g), and feed conversion ratio (1.686 *vs* 1.729), while MUL exhibited intermediate performance. At caecal level, MUH showed the lowest alpha diversity, a significantly different beta diversity, a reduction in Firmicutes, and a rise in Bacteroidetes, especially compared with MUL. MUH also exhibited a considerable decrease in Clostridiaceae and an overrepresentation of Bacteroidaceae and Lactobacillaceae. At blood level, MUH had lower hypoxanthine—probably due to its drop at caecal level—histidine, and uracil, while greater pyruvate, 2-oxoglutarate, and glucose. This study sheds light on the mode of action of this muramidase and lays the groundwork for future investigations on its effects on the intestinal ecosystem and systemic metabolism of broiler chickens.

## Introduction

In light of the gradual withdrawal of antibiotic growth promoters and increasing popularity of no-antibiotics-ever productions, the feed additive industry has been investing huge resources to provide poultry producers with effective and reliable gut-health-enhancers such as probiotics, prebiotics, organic acids, and enzymes^1,2^. Dietary enzymes have been demonstrated to boost feed digestibility by enriching the endogenous enzymatic repertoire of birds, monitor the proliferation of undesirable enteric bacteria, reduce gut mucosa irritation that would lead to inflammation, and promote the generation of a myriad of metabolites able to support gut health^1,3,4^. Lysozymes are renowned enzymes naturally produced by both prokaryotes and eukaryotes^5^. In animals, they are secreted via a number of body fluids, like tears, saliva, airway fluid, and breast milk, among others^6^. Avian lysozymes are mainly found in the egg albumen^5,6^. The chicken lysozyme—alias c-type or hen egg white lysozyme—was isolated for the first time by Sir Alexander Fleming and is referred to as the lysozyme per excellence^5^. Nile and colleagues^7^ also demonstrated that the small intestine enterocytes of chickens express lysozymes. Lysozymes play a pivotal role in the innate immune response of animals: they act as broad-spectrum antimicrobial—specifically bacteriolytic—proteins by hydrolyzing the β-(1,4)-glycosidic bond between *N*-acetylmuramic acid (NAM) and *N*-acetylglucosamine (NAG) residues of peptidoglycan (PGN), the major component of bacterial cell walls. Lysozymes are also known as muramidases because they are PGN *N*-acetylmuramoylhydrolase^5,6,8^. Muramidase-based feed additives have been shown to have positive effects on pigs^9–11^, rabbits^12^, and chickens^13,14^, which have predominantly been attributed to a modulation of the gastrointestinal (GI) microbiota^12–15^. Muramidases also possess immunomodulatory functions^16^ that have recently been confirmed in livestock^17–20^. A novel dietary muramidase, obtained through a biotech process^21^, has been shown to degrade PGN-containing bacteria cell debris. It is thought that the cleavage of luminal PGN can result in an optimization of digestive and absorptive functions as well as a positive modulation of the intestinal inflammatory response, with consequent improvements in gut health and performance of broiler chickens^22–24^. However, very little is currently known about the mechanisms of action of this muramidase. The present research sought to address this knowledge gap by studying performance traits, welfare indicators, breast muscle myopathies, caecal microbiome, and caecal and plasmatic metabolomes of broiler chickens supplemented with this dietary muramidase.

## Results

### Performance traits

**Figure 1:**
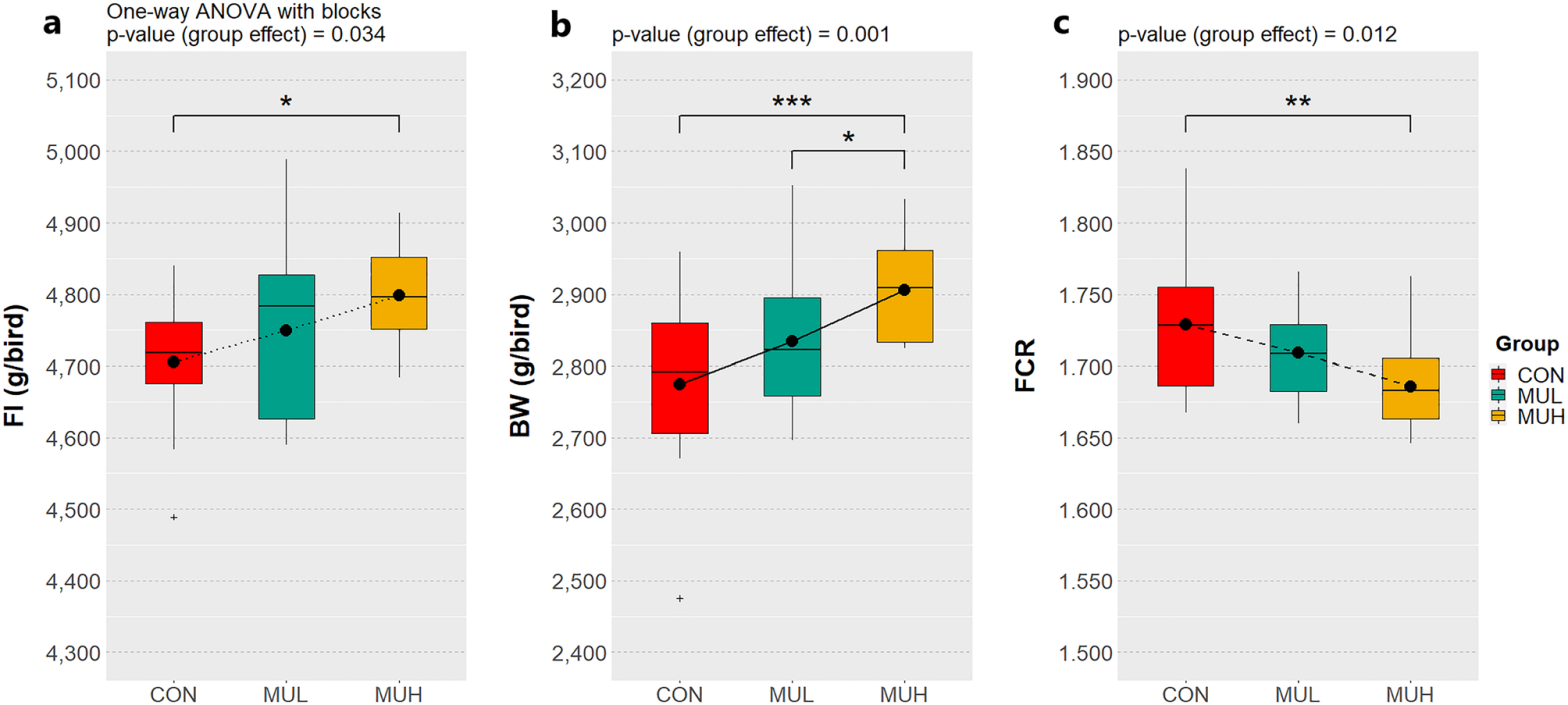
Cumulative FI (**a**), BW (**b**), and FCR (**c**) of CON, MUL, and MUH (0–42 d). FI, BW, and FCR were measured on 12 replicates per group arranged in a randomized complete block design. Group means are drawn as black dots inside the boxes. *p-value ≤ 0.05; **p-value < 0.01; ***p-value < 0.001. Linear trends are drawn as black lines connecting the group means (dotted line, *p*-value < 0.05; dashed line, *p*-value < 0.01; solid line, *p*-value < 0.001).

Chicks weighed approximately 42 g at placement with no inter-group significant differences. At the end of starter phase, MUL and MUH exhibited a higher BW than CON (199.5, 204.8, and 205.8 g for CON, MUL, and MUH, respectively; *p* < 0.05), whereas only MUH showed a lower FCR than CON (1.267 *vs* 1.240 for CON and MUH, respectively; *p* < 0.05) (Table 1). MUH reached the greatest BW at the conclusion of the first grower phase (765.3, 785.1, and 818.8 g for CON, MUL, and MUH, respectively; *p* < 0.05) as well as a higher FI than CON (866.0 and 907.7 g for CON and MUH, respectively; *p* < 0.05) (Table 1). At the end of the second grower phase, MUH showed the greatest BW (1,344, 1,375, and 1,443 g for CON, MUL, and MUH, respectively; *p* < 0.05) and lowest FCR (1.673, 1.651, and 1.590 for CON, MUL, and MUH, respectively; *p* < 0.05) (Table 1). At the conclusion of finisher phase, MUH exhibited a greater BW than CON (2,775 and 2,906 g for CON and MUH, respectively; *p* < 0.05) and MUL (2,835 and 2,906 g for MUL and MUH, respectively; *p* = 0.05), while other performance traits were unaffected (Table 1). Results of the overall trial indicate that, in addition to BW, MUH outperformed CON in terms of cumulative FI and FCR (4,705 g and 1.729, and 4,798 g and 1.686 for CON and MUH, respectively; *p* < 0.05) (Table 1; Fig. 1). Polynomial contrasts also revealed that cumulative FI and BW significantly increased while FCR decreased across groups in a linear fashion (Fig. 1). Lastly, mortality rate was not significantly influenced (Table 1).

**Table 1:** Performance traits of CON, MUL, and MUH at the end of each feeding phase and in the overall trial (0–42 d)^†^.

Trait	Group			SEM^‡^	*p*-value^‡^
	CON	MUL	MUH		
Chick weight (g)	42.18	42.11	42.20	0.08	0.685
**Starter (0–9 d)**					
BW (g/bird)	199.5^b^	204.8^a^	205.8^a^	1.29	0.005
DWG (g/bird/d)^§^	17.48^b^	18.08^a^	18.18^a^	0.15	0.005
DFI (g/bird/d)^§^	22.15	22.61	22.53	0.14	0.053
FI (g/bird)^§^	199.3	203.5	202.8	1.21	0.053
FCR^§^	1.267^a^	1.251^ab^	1.240^b^	0.01	0.016
Mortality (%)	0.00	0.00	0.00	-	-
**Grower I (10–21 d)**					
BW (g/bird)	765.3^b^	785.1^b^	818.8^a^	6.41	< 0.001
DWG (g/bird/d)^§^	47.15^b^	48.36^b^	51.10^a^	0.48	< 0.001
DFI (g/bird/d)^§^	72.17^b^	73.22^ab^	75.65^a^	0.94	0.044
FI (g/bird)^§^	866.0^b^	878.6^ab^	907.7^a^	11.30	0.044
FCR^§^	1.532	1.515	1.482	0.02	0.108
Mortality (%)	0.00	0.00	0.13	0.01	0.384
**Grower II (22–28 d)**					
BW (g/bird)	1,344^b^	1,375^b^	1,443^a^	11.50	< 0.001
DWG (g/bird/d)^§^	82.69^b^	83.88^b^	89.13^a^	1.07	0.001
DFI (g/bird/d)^§^	138.0	138.3	141.6	1.14	0.062
FI (g/bird)^§^	966.0	968.0	991.5	7.96	0.062
FCR^§^	1.673^a^	1.651^a^	1.590^b^	0.01	0.001
Mortality (%)	0.26	0.39	0.26	0.02	0.877
**Finisher (29–42 d)**					
BW (g/bird)	2,775^b^	2,835^b^	2,906^a^	20.10	0.001
DWG (g/bird/d)^§^	101.6	103.5	103.2	0.85	0.235
DFI (g/bird/d)^§^	191.0	192.8	192.6	0.95	0.349
FI (g/bird)^§^	2,674	2,699	2,696	13.30	0.349
FCR^§^	1.883	1.864	1.866	0.01	0.547
Mortality (%)	1.41	1.41	1.80	0.02	0.948
**Overall (0–42 d)**					
BW (g/bird)	2,775^b^	2,835^b^	2,906^a^	20.10	0.001
DWG (g/bird/d)^§^	65.03^b^	66.46^ab^	68.13^a^	0.48	0.001
DFI (g/bird/d)^§^	111.8^b^	112.8^ab^	114.0^a^	0.58	0.043
FI (g/bird)^§^	4,705^b^	4,749^ab^	4,798^a^	23.50	0.034
FCR^§^	1.729^a^	1.709^ab^	1.686^b^	0.01	0.012
Mortality (%)	1.68	1.80	2.18	0.02	0.746

^†^Mean values of 12 replicates per group arranged in a randomized complete block design.

^‡^Reported values refer to the experimental factor group.

^§^Corrected for mortality.

^a, b^Within a row, means with no common superscripts differ significantly (*p*-value ≤ 0.05).

SEM: Standard error of the mean.

### Processing yields

Data of carcass and cut-up yields—generated at processing in a commercial plant—were not subjected to statistical analysis because measured on a group basis. Notwithstanding, the considerable sample size (i.e., more than 750 observations/group) made it possible to notice that muramidase-supplemented groups had a greater eviscerated carcass yield (70.1, 70.4, and 70.8% for CON, MUL, and MUH, respectively) and breast yield calculated as percentage of eviscerated carcass weight (30.6, 30.9, and 31.3% for CON, MUL, and MUH, respectively).

### Muramic acid concentration in excreta samples

Concentration of muramic acid (total, soluble, and their ratio), used as a measure for the concentration of hydrolyzed bacterial PGN in excreta samples, is given in Table 2. In every feeding phase, muramidase-supplemented groups exhibited a significantly higher soluble fraction of muramic acid as well as a greater ratio with respect to total muramic acid than CON. Moreover, it was found a weak negative Pearson’s correlation coefficient between soluble to total muramic acid ratio and FCR (*r* = −0.30; *p* < 0.001).

**Table 2:** Total muramic acid, soluble muramic acid, and their ratio found in excreta samples of CON, MUL, and MUH at the end of each feeding phase^†^.

Variable	Group			SEM^‡^	*p*-value^‡^
	CON	MUL	MUH		
**Starter (0–9 d)**					
Total muramic acid (mg/kg)	497.8	398.8	444.0	54.7	0.453
Soluble muramic acid (mg/kg)	121.7^b^	214.6^a^	256.6^a^	19.0	< 0.001
Soluble/total muramic acid (%)	25.3^b^	55.6^a^	62.2^a^	3.2	< 0.001
**Grower I (10–21 d)**					
Total muramic acid (mg/kg)	932.2^a^	747.0^b^	897.6^a^	37.4	0.005
Soluble muramic acid (mg/kg)	284.6^b^	575.7^a^	539.8^a^	31.3	< 0.001
Soluble/total muramic acid (%)	30.8^c^	78.5^a^	62.8^b^	3.6	< 0.001
**Grower II (22–28 d)**					
Total muramic acid (mg/kg)	660.4	666.1	648.7	38.1	0.947
Soluble muramic acid (mg/kg)	148.2^b^	325.3^a^	335.6^a^	27.6	< 0.001
Soluble/total muramic acid (%)	23.5^b^	49.6^a^	51.2^a^	2.7	< 0.001
**Finisher (29–42 d)**					
Total muramic acid (mg/kg)	830.6^a^	606.3^b^	740.4^ab^	58.6	0.041
Soluble muramic acid (mg/kg)	127.6^b^	272.7^a^	284.4^a^	20.2	< 0.001
Soluble/total muramic acid (%)	17.1^b^	47.3^a^	40.9^a^	4.1	< 0.001

^†^Mean values of 12 replicates per group arranged in a randomized complete block design.

^‡^Reported values refer to the experimental factor group.

^a, b^Within a row, means with no common superscripts differ significantly (*p*-value < 0.05).

SEM: Standard error of the mean.

### Incidence and severity of foot-pad dermatitis and breast muscle myopathies

**Figure 2:**
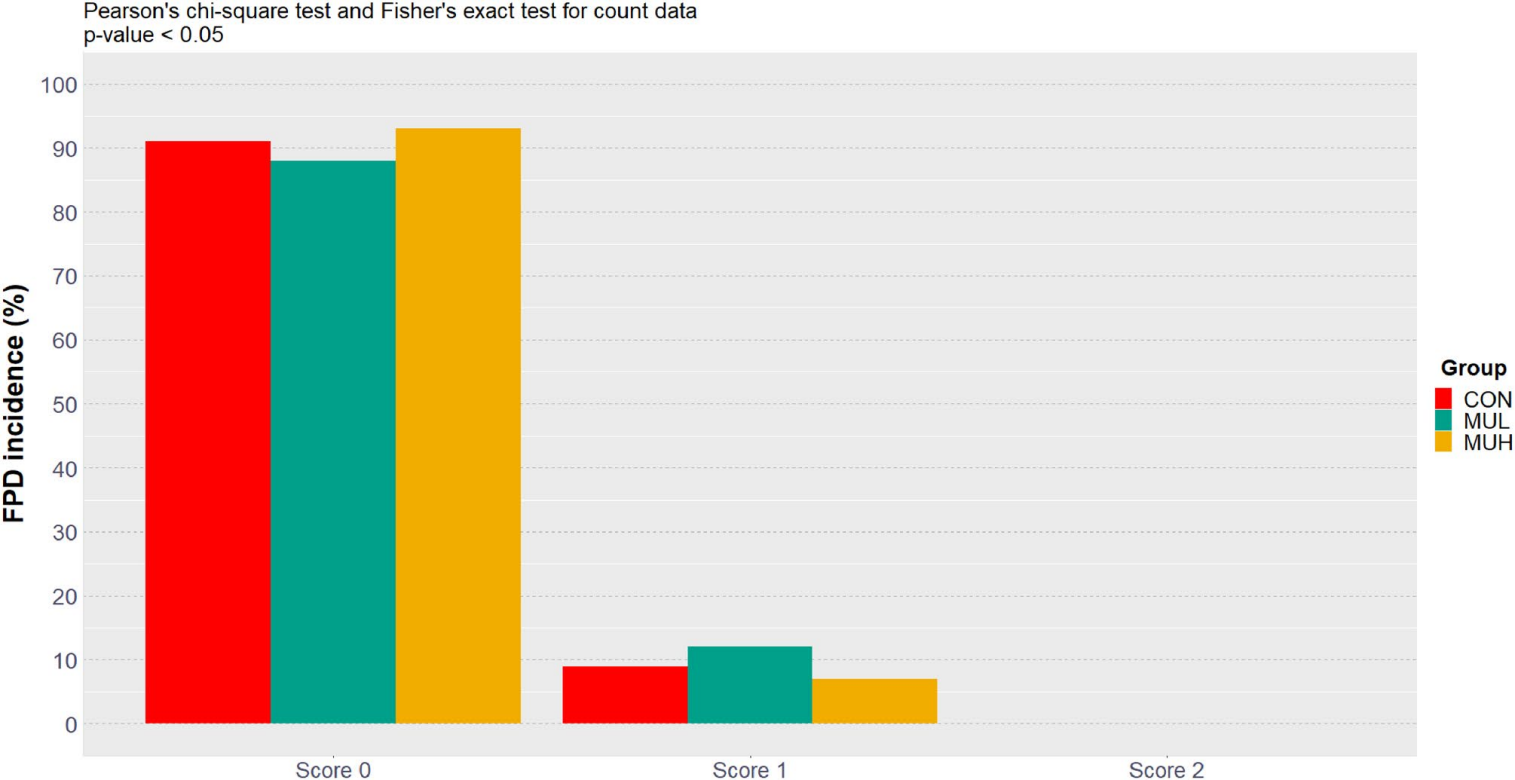
FPD incidence and severity (score 0, no lesions; score 1, mild lesions; score 2, severe lesions) of CON, MUL, and MUH at slaughter (42 d). FPD were macroscopically measured on 1 foot per bird (*n* = 675, 673, and 684 for CON, MUL, and MUH, respectively).

**Figure 3:**
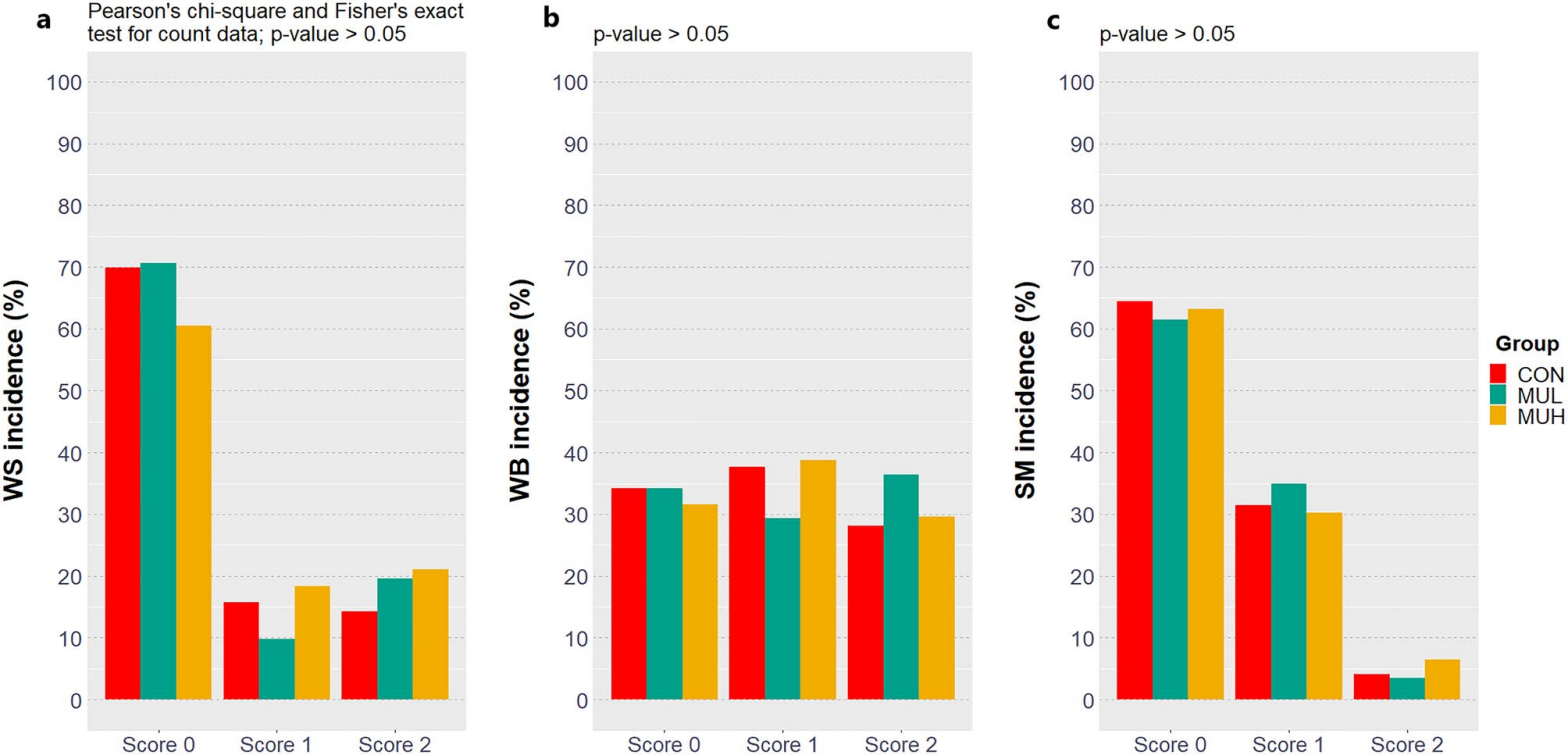
WS (**A**), WB (**B**), and SM (**C**) incidence and severity (score 0, no abnormalities; score 1, moderate degree; score 2, severe degree) of CON, MUL, and MUH at slaughter (42 d). WS, WB, and SM were macroscopically measured on a representative sample of breast fillets (*n* = 146, 143, and 152 for CON, MUL, and MUH, respectively) after chilling, deboning, and skin removal.

Occurrence of foot-pad dermatitis (FPD) was significantly associated with the factor group (Fig. 2). MUH was 0.58 times less likely to develop FPD than MUL; that is, supplementing chickens with muramidase at high dose decreased by 42% the relative risk of developing FPD compared to their counterparts supplemented at low dose. However, MUL diet tended to increase by 33% the relative risk of FPD development compared to CON diet (Table 3). On the other hand, breast muscle myopathies did not show a significant relationship with the factor group (Fig. 3).

**Table 3:** FPD risk ratio computation on 2 by 2 tables aligning the combinations of levels of the factor group and putting binarily aggregated FPD scores in columns.

Contrast^†^	Risk ratio^‡^	Relative risk of FPD development^§^	*p*-value^¤^
MUL *vs* CON	1.33 (0.97, 1.83)	+ 33%	0.070
MUH *vs* CON	0.78 (0.54, 1.12)	-22%	0.170
MUH *vs* MUL	0.58 (0.41, 0.82)	-42%	< 0.001

^†^The inverse contrasts produce a risk ratio equals to the reciprocal of the risk ratio shown.

^‡^95% Wald confidence interval is given in brackets.

^§^Calculated as risk ratio minus 1 percentagewise.

^¤^Count data were statistically analyzed via Pearson’s chi-square test.

### Caecal microbiome

**Figure 4:**
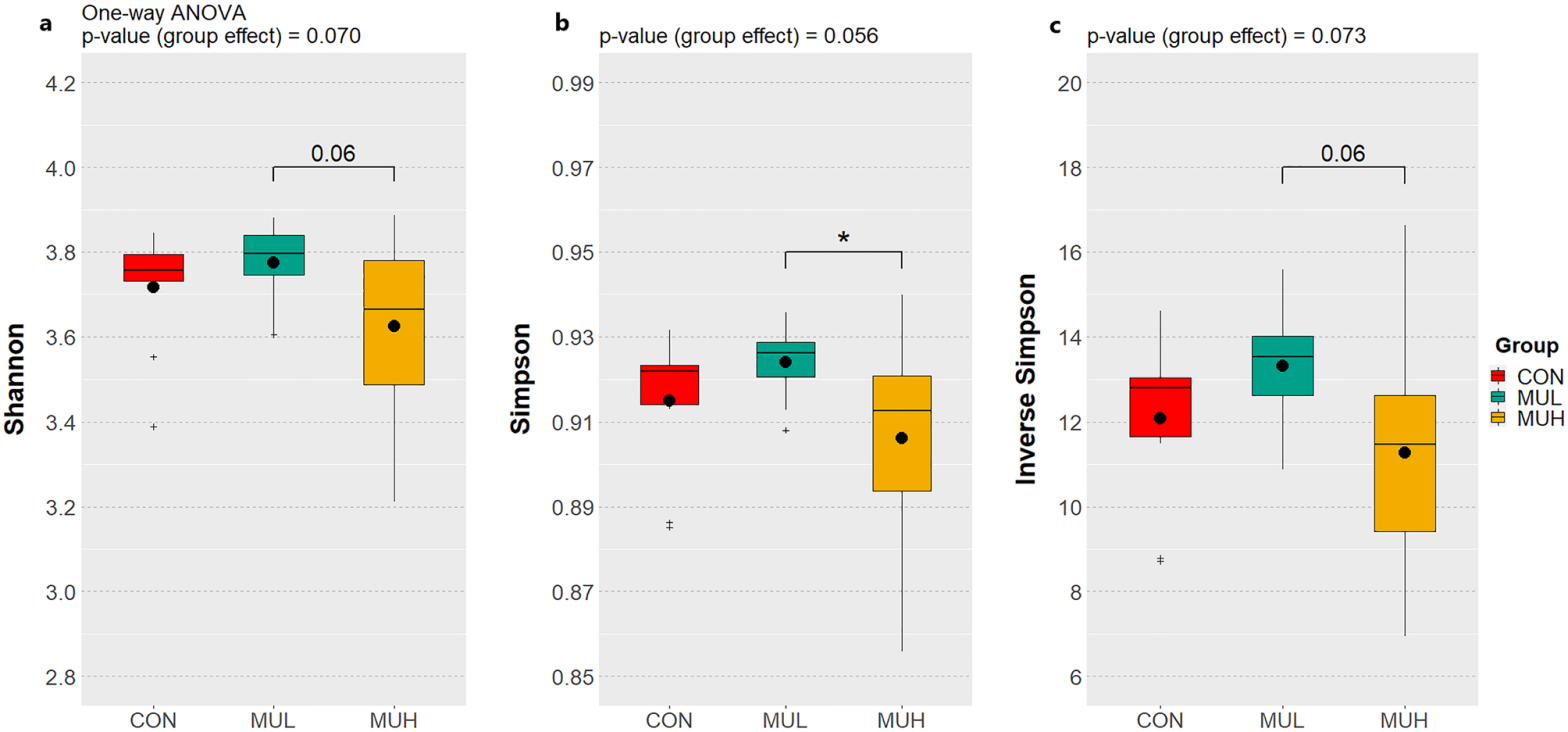
Shannon (**a**), Simpson (**b**), and Inverse Simpson (**c**) alpha diversity of CON, MUL, and MUH caecal contents at slaughter (42 d). Group means are drawn as black dots inside the boxes. *p-value < 0.05.

**Figure 5:**
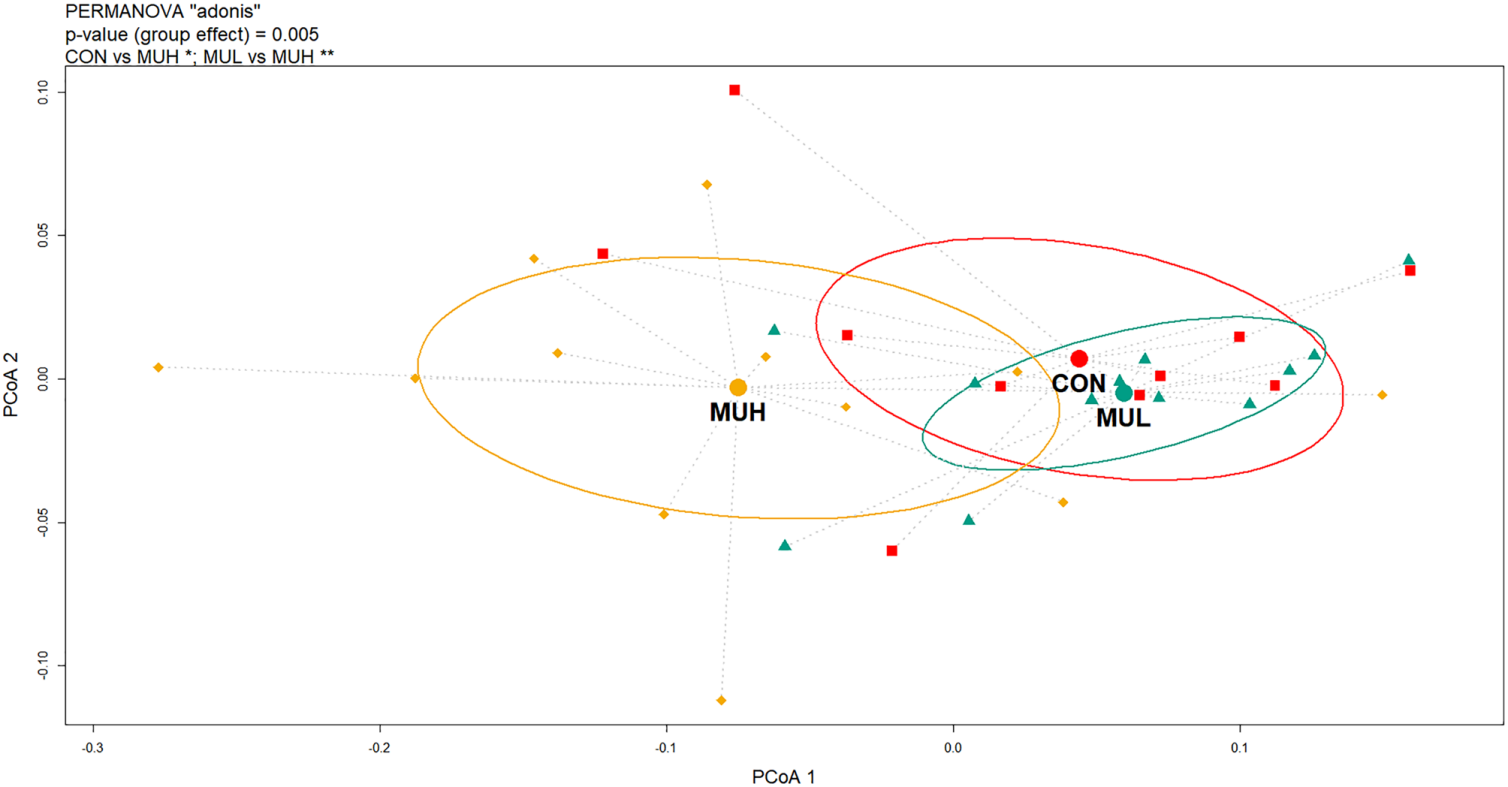
PCoA plot based on Bray–Curtis distance matrix used to compute beta diversity between caecal content samples of CON (red squares), MUL (green triangles), and MUH (yellow diamonds) at slaughter (42 d). The large, colored dots are the group centroids, while the colored plane curves are the standard deviational ellipses.

**Figure 6:**
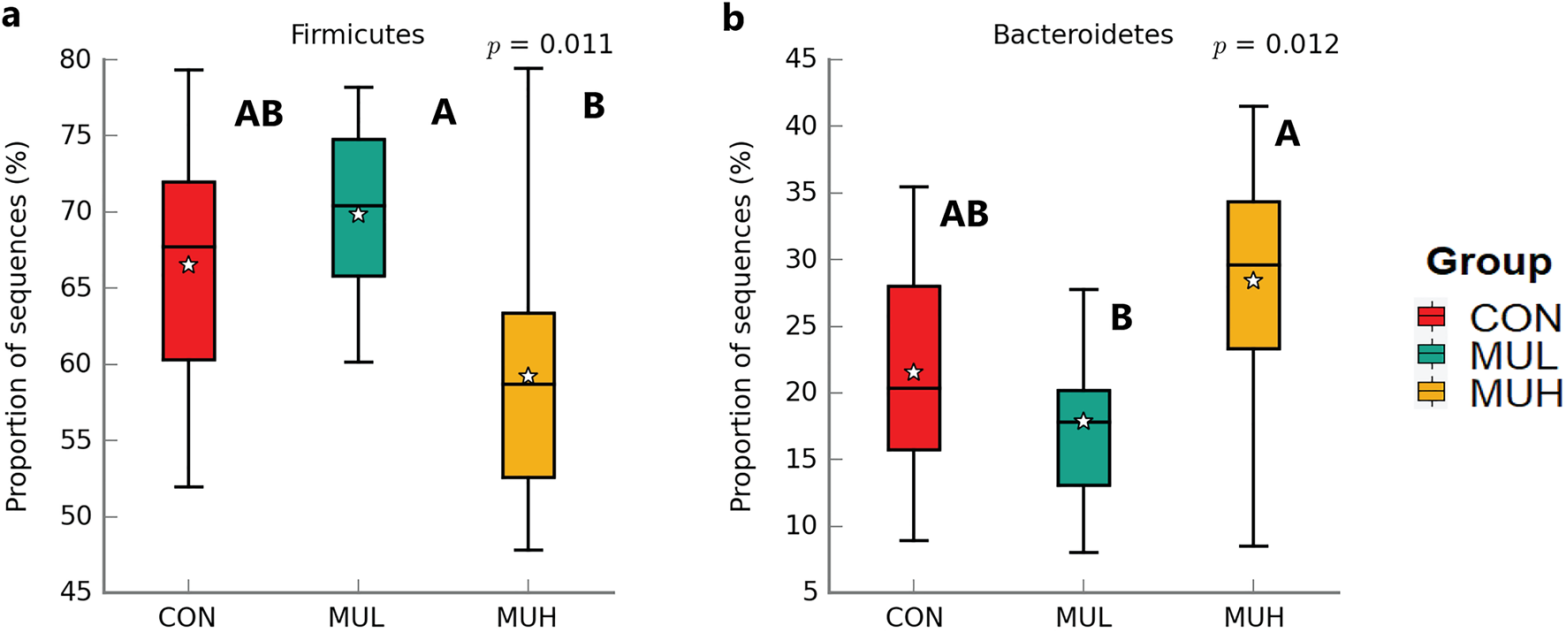
Relative abundance of caecal Firmicutes (**a**) and Bacteroidetes (**b**) of CON, MUL, and MUH at slaughter (42 d). Group means are drawn as white stars inside the boxes. Kruskal–Wallis H-test *p*-values are reported in the top-right corner. A, B: *p*-value < 0.01.

**Figure 7:**
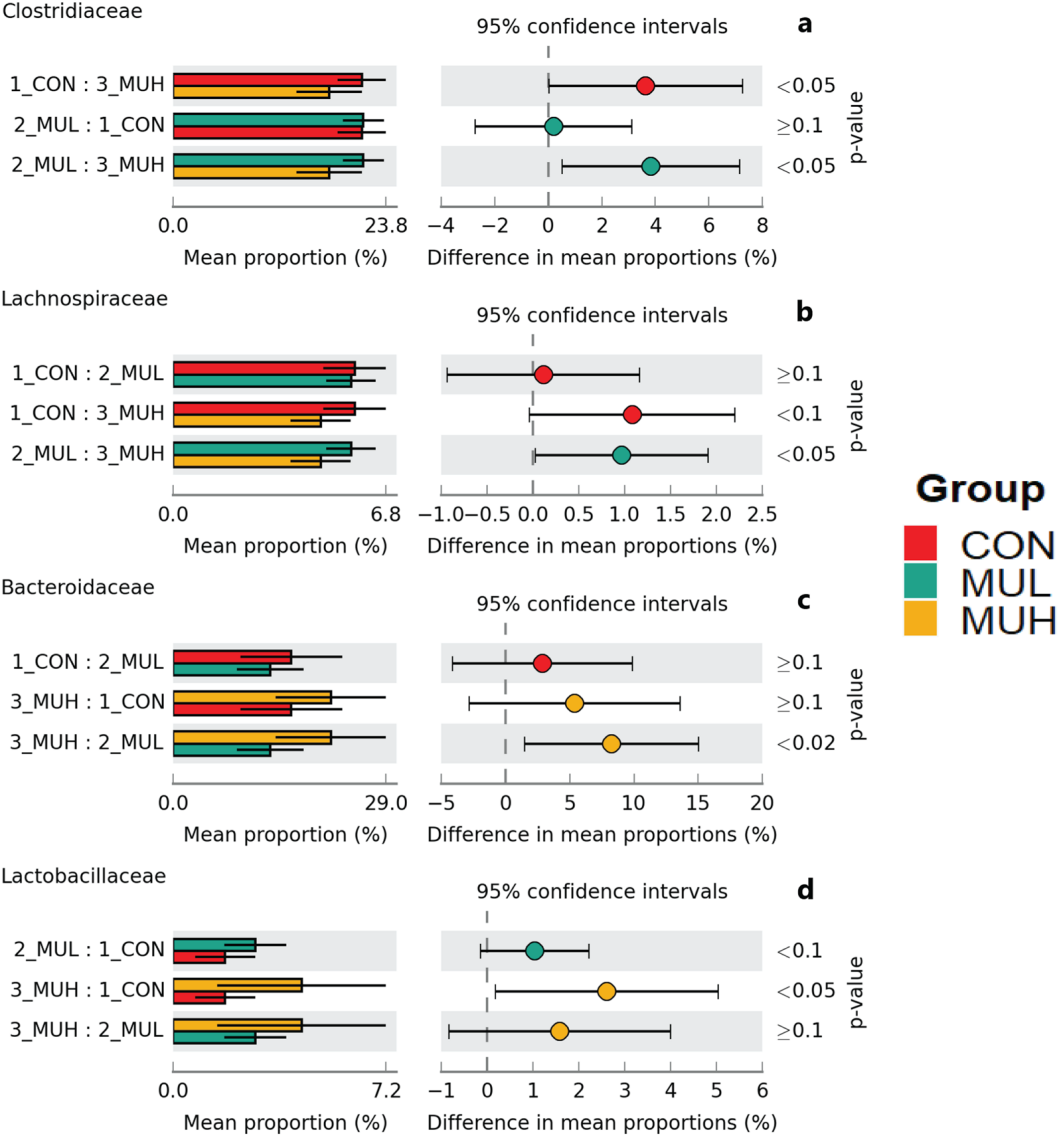
Extended error bar plots of mean relative abundance differences of caecal Clostridiaceae (**a**), Lachnospiraceae (**b**), Bacteroidaceae (**c**), and Lactobacillaceae (**d**) between CON, MUL, and MUH at slaughter (42 d). Colored bars and black lines indicate the mean relative abundance and standard deviation of the bacterial family, respectively (left side). Colored dots inside the 95% confidence intervals signify the difference of mean relative abundance of the bacterial family for each pairwise comparison (center). Games-Howell post-hoc test *p*-values are reported (right side).

**Figure 8:**
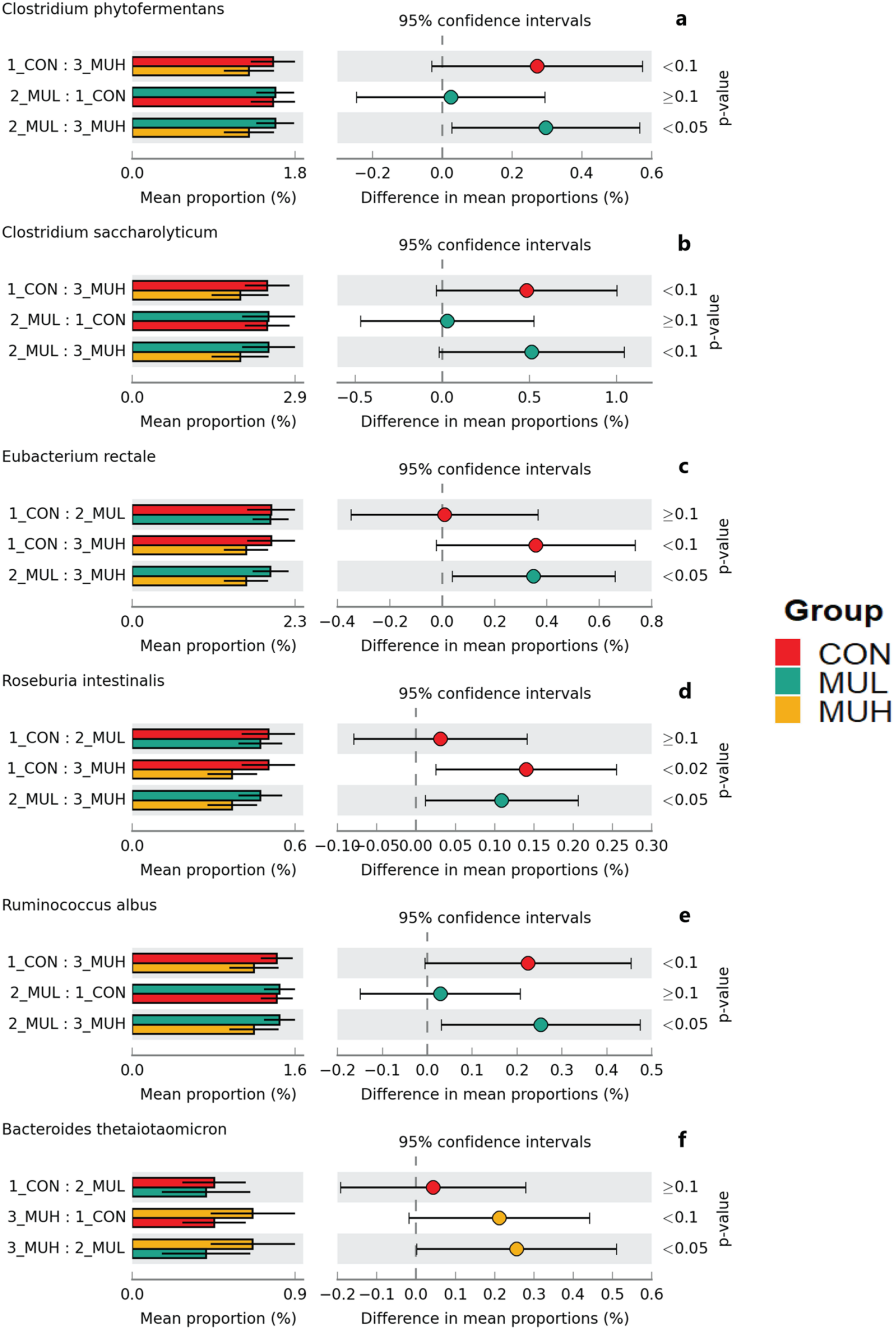
Extended error bar plots of mean relative abundance differences of caecal *C. phytofermentans* (**a**), *C. saccharolyticum* (**b**), *E. rectale* (**c**), *R. intestinalis* (**d**), *R. albus* (**e**), and *B. thetaiotaomicron* (**f**) between CON, MUL, and MUH at slaughter (42 d). Colored bars and black lines indicate the mean relative abundance and standard deviation of the bacterial species, respectively (left side). Colored dots inside the 95% confidence intervals signify the difference of mean relative abundance of the bacterial species for each pairwise comparison (center). Games-Howell post-hoc test *p*-values are reported (right side).

**Figure 9:**
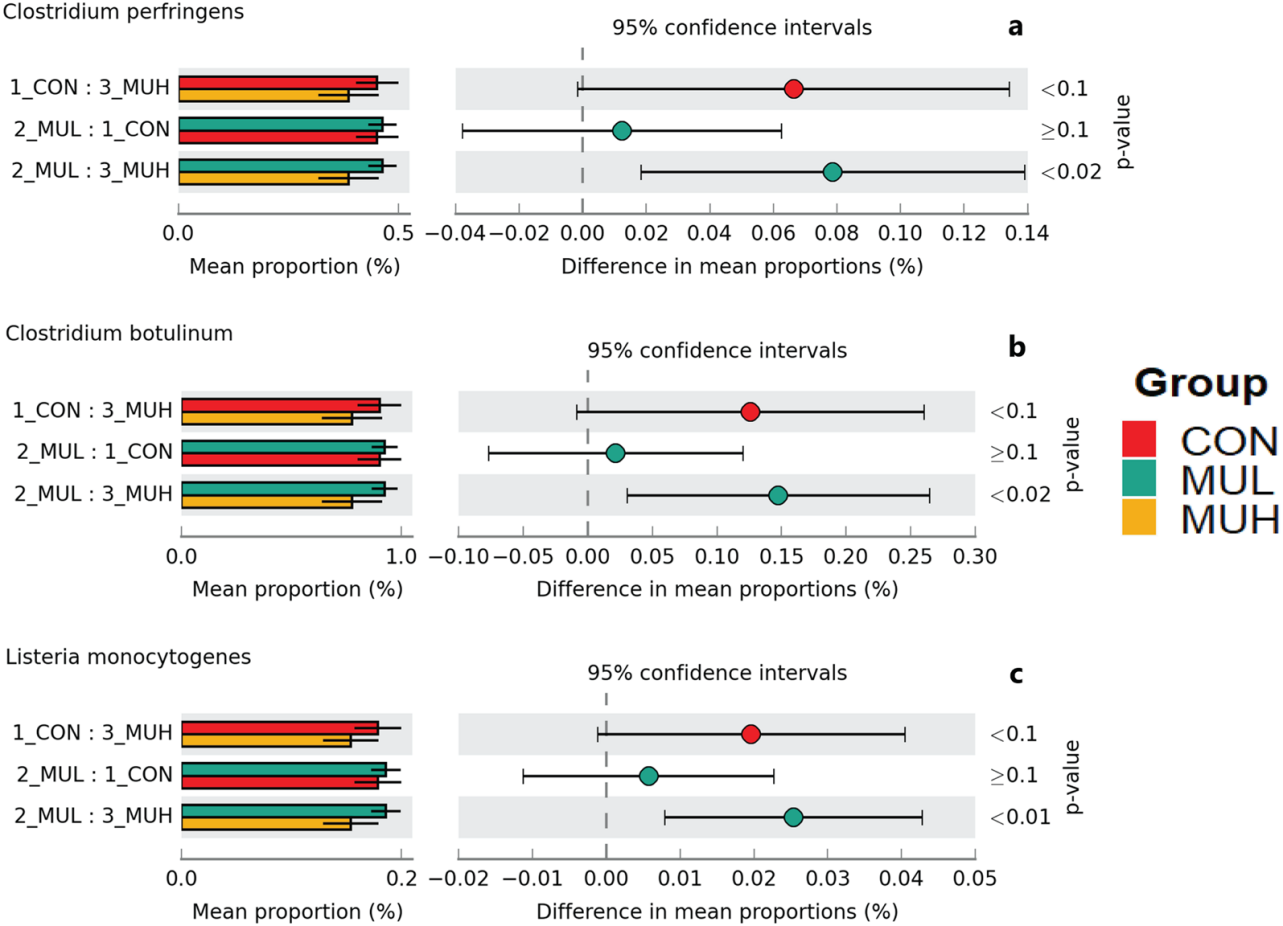
Extended error bar plots of mean relative abundance differences of caecal *C. perfringens* (**a**), *C. botulinum* (**b**), and *L. monocytogenes* (**c**) between CON, MUL, and MUH at slaughter (42 d). Colored bars and black lines indicate the mean relative abundance and standard deviation of the bacterial species, respectively (left side). Colored dots inside the 95% confidence intervals signify the difference of mean relative abundance of the bacterial species for each pairwise comparison (center). Games-Howell post-hoc test *p*-values are reported (right side).

**Figure 10:**
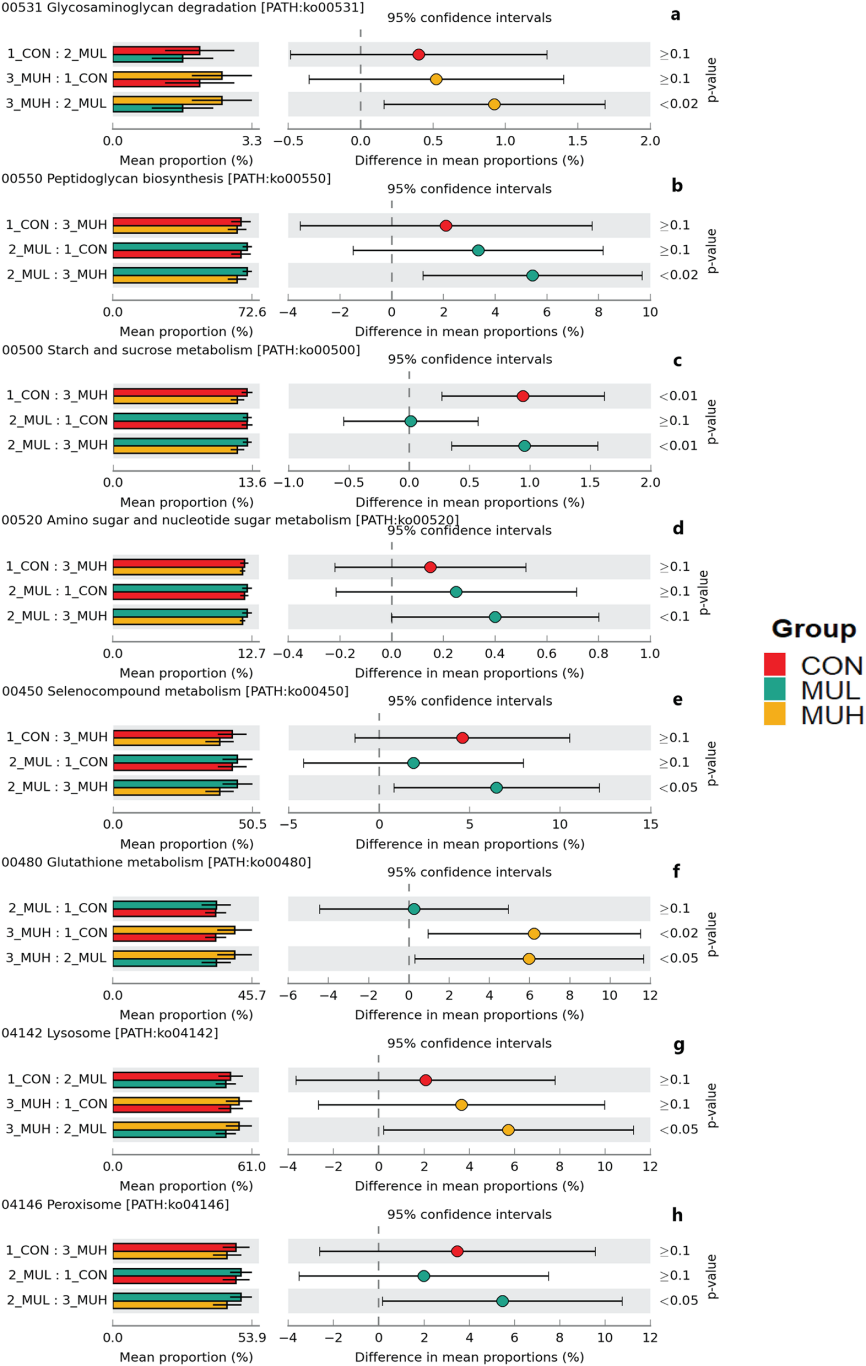
Extended error bar plots of mean relative abundance differences of glycan biosynthesis and metabolism (**a**, **b**), carbohydrate metabolism (**c, d**), metabolism of other amino acids (**e, f**), and transport and catabolism (**g, h**) between caecal samples of CON, MUL, and MUH at slaughter (42 d). Colored bars and black lines indicate the mean relative abundance and standard deviation of the KEGG level 3 path, respectively (left side). Colored dots inside the 95% confidence intervals signify the difference of mean relative abundance of the KEGG level 3 path for each pairwise comparison (center). Games-Howell post-hoc test *p*-values are reported (right side).

MUH exhibited a lower alpha diversity than MUL (*p* < 0.05 for Simpson and *p* = 0.06 for Shannon and Inverse Simpson, respectively), while CON did not differ from muramidase-supplemented groups (Fig. 4). Regarding beta diversity, the PCoA revealed an evident segregation of MUH samples (Fig. 5). Besides this visual distinction, the PERMANOVA confirmed a group effect on beta diversity (*p* = 0.005, R^2^ = 0.22), while the pairwise permutation MANOVA revealed a significant differentiation of MUH compared to other groups. At phylum level, MUH showed a lower relative abundance of Firmicutes and a greater of Bacteroidetes than MUL (69.9% and 17.9%, and 59.2% and 28.4% for MUL and MUH, respectively; *p* < 0.05) (Fig. 6). Similarly, the Firmicutes to Bacteroidetes ratio differed between MUH and MUL (2.7 and 4.5, respectively; *p* = 0.05). At family level, Clostridiaceae were underrepresented in MUH compared to other groups (21.1, 21.3, and 17.4% for CON, MUL, and MUH, respectively; *p* < 0.05) and Lachnospiraceae were less abundant in MUH than MUL (5.7% and 4.7% for MUL and MUH, respectively; *p* < 0.05). Contrariwise, MUH exhibited a higher relative abundance of Bacteroidaceae than MUL (13.2% and 21.5% for MUL and MUH, respectively; *p* < 0.02). Relative abundance of Lactobacillaceae was greater in MUH than CON (4.4% and 1.8%, respectively; *p* < 0.05) (Fig. 7). Lastly, at species level, relative abundance of *C. phytofermentans*, *C. saccharolyticum*, *C. cellulolyticum*, and *C. butyricum* was lower in MUH than other groups (Fig. 8). This variation also applies to *Eubacterium rectale*, *Roseburia intestinalis*, *Ruminococcus albus*, *C. perfringens*, *C. botulinum*, and *Listeria monocytogenes* (Fig. 8–9). An opposite pattern, however, was observed for *Bacteroides thetaiotaomicron* whose relative abundance was higher in MUH than MUL (*p* < 0.05) (Fig. 8). Relative abundance of genes that significantly differed between groups is shown in Fig. 10. At glycan biosynthesis and metabolism level, MUH showed a greater relative abundance of genes associated to glycosaminoglycan degradation pathway than MUL (*p* < 0.02), while relative abundance of genes involved in peptidoglycan biosynthesis pathway was affected in the opposite fashion (*p* < 0.02). Genes involved in starch and sucrose metabolism and amino sugar and nucleotide metabolism were affected by the factor group: the former had a higher relative abundance in CON and MUL than MUH (*p* < 0.01), whereas the latter showed an increasing trend in MUL compared to MUH (*p* < 0.1). Genes involved in seleno compound metabolism pathway showed an increase in MUL compared to MUH (*p* < 0.05). However, MUH exhibited a higher relative abundance of genes involved in glutathione metabolism pathway than both CON and MUL (*p* < 0.02 and *p* < 0.05, respectively). Lastly, two transport and catabolism pathways revealed an inter-group difference: genes involved in lysosome path had a greater relative abundance in MUH than MUL (*p* < 0.05), while genes linked to peroxisome path varied the other way around (*p* < 0.05).

### Plasma and caecal content metabolomes

**Figure 11:**
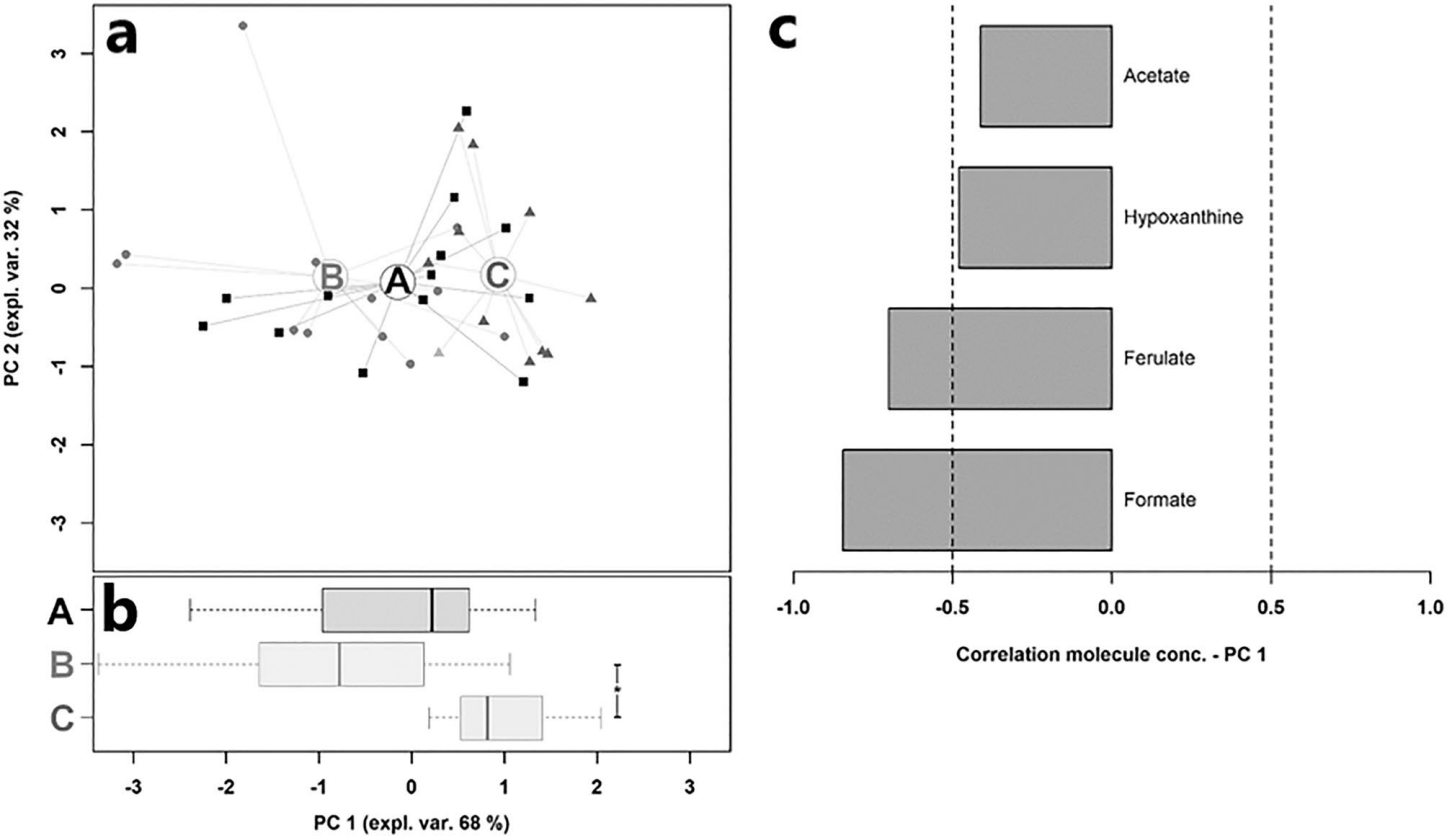
rPCA model on caecal metabolites of Table 4. In the score plot (**a**), samples of CON (“A”), MUL (“B”), and MUH (“C”) are drawn as squares, circles, and triangles, respectively. Wide circles are the group medians. The box plot (**b**) summarizes the position of samples along PC1. The loading plot (**c**) reports the correlations between the concentration of each metabolite and its importance over PC1. Grey bars indicate significant correlations (*p*-value < 0.05).

**Figure 12:**
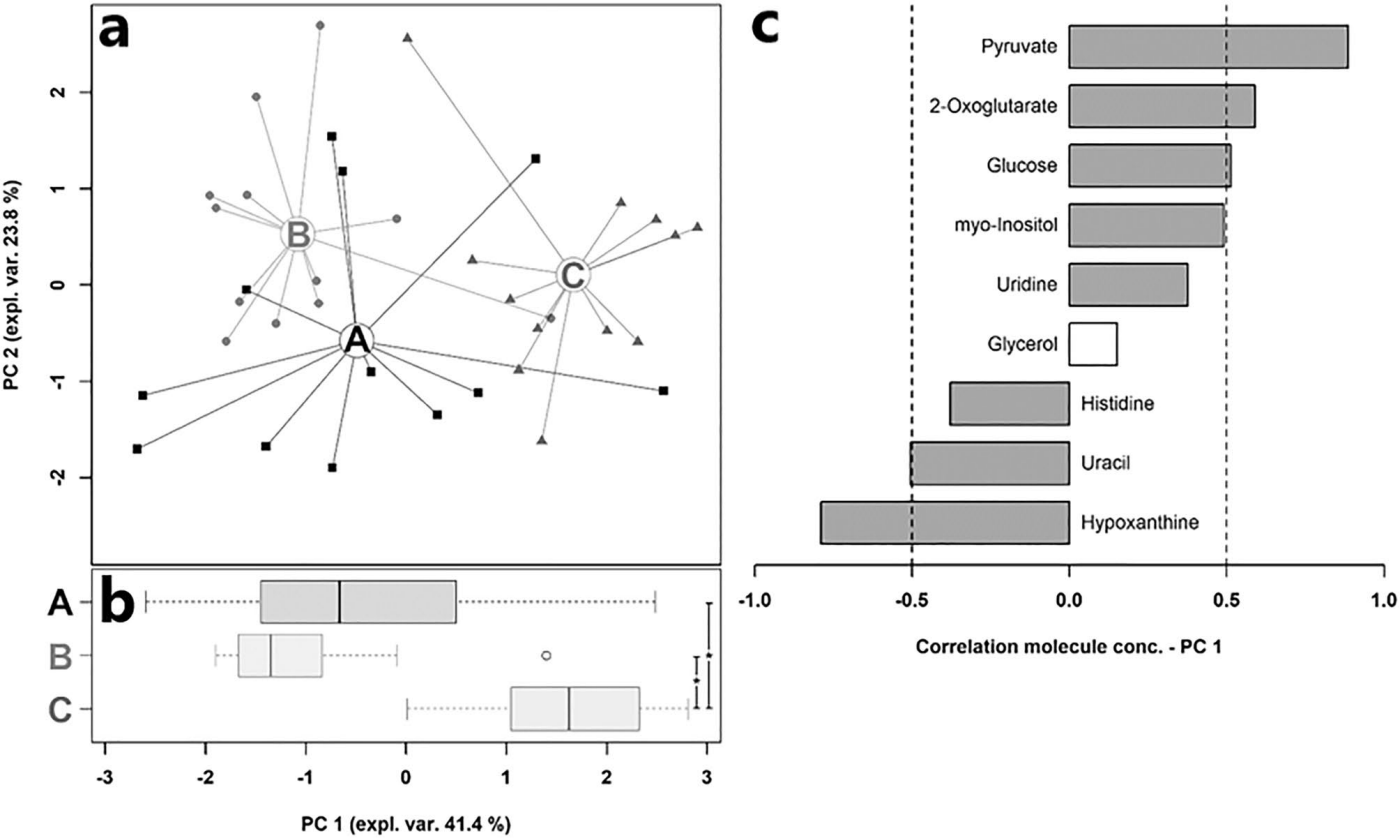
rPCA model on plasma metabolites of Table 5. In the score plot (**a**), samples of CON (“A”), MUL (“B”), and MUH (“C”) are drawn as squares, circles, and triangles, respectively. Wide circles are the group medians. The box plot (**b**) summarizes the position of samples along PC1. The loading plot (**c**) reports the correlations between the concentration of each metabolite and its importance over PC1. Grey bars indicate significant correlations (*p*-value < 0.05).

Plasma and caecal content ^1^H-NMR spectra were registered and 54 and 78 molecules were assigned and quantified, respectively. At caecal content level, the concentration of 4 metabolites showed a significant inter-group difference (Table 4). While acetate, ferulate, and formate were greater in MUL than MUH (*p* < 0.05), hypoxanthine was higher in CON than MUH (*p* < 0.05). The rPCA model shown in Fig. 11 was built on these molecules. The principal component one (PC1) accounts for 68% of the variance explained by the model and summarizes the differences between groups. PC1 scores of MUH samples are markedly or marginally higher than those of other groups, resulting in a group-based clustering of samples mainly led by ferulate and formate (*r* < −0.5). At plasma level, the concentration of 9 metabolites showed a significant variation between groups (Table 5). Specifically, a higher concentration of pyruvate was observed in MUH than CON and MUL (*p* < 0.05), while 2-oxoglutarate, glucose, and uridine were greater in MUH than MUL (*p* < 0.05). On the other hand, MUH showed a higher concentration of myo-inositol than CON (*p* < 0.05), whereas CON exhibited a greater concentration of histidine than MUH (*p* < 0.05). Similarly, both CON and MUL had a higher concentration of hypoxanthine than MUH (*p* < 0.05), while MUL showed a greater uracil concentration than MUH (*p* < 0.05). Figure 12 illustrates the rPCA model produced as described above. Samples of MUH are characterized by higher PC1 scores and distinctly segregate (*p* < 0.05): this separation is predominantly driven by pyruvate, 2-oxoglutarate, glucose, uracil, and hypoxanthine (*r* > 0.5 or < −0.5).

**Table 4:** Caecal metabolites showing significantly different concentrations (mmol/L) between CON, MUL, and MUH at slaughter (42 d)^†^.

Metabolite	Group		
	CON	MUL	MUH
Acetate	4.70E-02 (2.79E-02)^ab^	6.38E-02 (1.65E-02)^a^	4.82E-02 (1.81E-02)^b^
Hypoxanthine	6.67E-05 (3.71E-05)^a^	5.46E-05 (3.91E-05)^ab^	3.22E-05 (2.05E-05)^b^
Ferulate	6.54E-05 (1.64E-05)^ab^	8.04E-05 (2.99E-05)^a^	5.16E-05 (2.75E-05)^b^
Formate	7.17E-05 (1.52E-05)^ab^	7.86E-05 (2.65E-05)^a^	6.08E-05 (1.38E-05)^b^

^†^Table entries are means and standard deviation in brackets.

^a, b^Within a row, means with no common superscripts differ significantly (*p*-value < 0.05).

**Table 5:** Plasma metabolites showing significantly different concentrations (mmol/L) between CON, MUL, and MUH at slaughter (42 d)^†^.

Metabolite	Group		
	CON	MUL	MUH
Pyruvate	4.97E-02 (1.92E-02)^b^	4.19E-02 (8.92E-03)^b^	6.45E-02 (2.50E-02)^a^
2-Oxoglutarate	7.31E-03 (2.17E-03)^ab^	6.07E-03 (2.60E-03)^b^	8.97E-03 (2.61E-03)^a^
Glucose	4.73E + 00 (3.20E-01)^ab^	4.76E + 00 (4.69E-01)^b^	5.12E + 00 (3.75E-01)^a^
myo-Inositol	1.13E-01 (2.27E-02)^b^	1.10E-01 (1.66E-02)^ab^	1.29E-01 (2.42E-02)^a^
Uridine	4.36E-03 (1.90E-03)^ab^	3.26E-03 (1.13E-03)^b^	5.40E-03 (1.38E-03)^a^
Glycerol	2.11E-02 (4.29E-03)^b^	2.63E-02 (4.01E-03)^a^	2.33E-02 (6.32E-03)^ab^
Histidine	2.17E-02 (6.97E-03)^a^	2.09E-02 (5.59E-03)^ab^	1.97E-02 (4.91E-03)^b^
Uracil	3.37E-03 (9.47E-04)^ab^	4.27E-03 (8.40E-04)^a^	3.33E-03 (4.25E-04)^b^
Hypoxanthine	4.49E-03 (3.35E-03)^a^	5.47E-03 (2.02E-03)^a^	2.83E-03 (1.70E-03)^b^

^†^Table entries are means and standard deviation in brackets.

^a, b^Within a row, means with no common superscripts differ significantly (*p*-value < 0.05).

## Discussion

The purpose of this multi-omics investigation was to shed light on the mechanisms of action of a dietary muramidase supplemented to broiler chickens. MUH, the experimental group receiving the muramidase at high dose (i.e., 45,000 LSU(F)/kg feed), significantly outperformed the control group CON in terms of cumulative FI, BW, and FCR at 42 d. On the other hand, MUL, the low-dose group (i.e., 25,000 LSU(F)/kg feed), showed intermediate cumulative performance and did not differ from CON in a significant manner. It is worth highlighting that cumulative FI, BW, and FCR improved proportionately with muramidase dose. These results broadly support those of previous research assessing the administration of the same muramidase to broiler chickens, wherein birds supplemented at high inclusion levels (i.e., 35–45,000 LSU(F)/kg feed) performed better than their control and low-dose counterparts^21–24^. The non-invasive technique employed here to measure the muramidase-mediated PGN hydrolysis in excreta samples can be a reliable alternative to the ex vivo analysis lately illustrated by Frederiksen and co-workers^25^. Indeed, the proposed method confirmed that this muramidase effectively hydrolyzes bacterial PGN causing the release of fragments that, according to recent reports, can support intestinal health and performance of broiler chickens^22–24^. The observed reduction in FPD occurrence associated with muramidase supplementation, especially at high dose, is consistent with results obtained by Pirgozliev and colleagues^24^. Poultry foot-pad welfare greatly depends on litter quality (e.g., moisture and ammonia levels) and its management^26^. Although measuring litter parameters was beyond the scope of this work, a better nutrient utilization as well as less watery excreta—both commonly resulting from enhanced feed efficiency—may have played key roles in FDP risk attenuation^27^. Analysis of breast fillets revealed that the muramidase did not affect WS, WB, and SM. Therefore, under our experimental settings, this muramidase was able to improve growth performance without exerting negative effects on the occurrence of breast muscle myopathies, with positive implications for the sustainability of poultry meat production. Feeding trials on pigs^10,28–31^, rabbits^12^, and broiler chickens^13–15^ suggest that the beneficial effects of dietary muramidases can be ascribed to a modulatory activity on the GI microbiota. In the present investigation, the muramidase supplemented at high dose produced a drop in caecal alpha diversity. This result is in agreement with those of previous studies on pigs^29^ and, above all, on the same supplement fed to broiler chickens^19,23^. Moreover, MUH exhibited a different bacterial community structure at caecal level, especially compared to MUL. This is in accord with research on piglets and lactating sows^29,30^, and supports findings obtained in broiler chickens treated with the same muramidase^19^. Not only was changed the overall caecal bacterial community structure, but also its taxonomic composition. The observed underrepresentation of Firmicutes and outgrowth of Bacteroidetes, particularly evident for the comparison between MUH and MUL, seem to be consistent with results of Maga and colleagues^28^. These researchers fed pigs with the milk produced by transgenic goats expressing the human muramidase and proved that, in fecal samples of treated pigs, abundance of Firmicutes fell whereas that of Bacteroidetes raised over time. In the current study, MUH also showed a significant decrease in the Firmicutes to Bacteroidetes ratio compared with MUL. A considerable amount of papers has been published on the role played by the Firmicutes to Bacteroidetes ratio in the microbiota-to-host energy supply and development of obesity. However, since contradictory outcomes are not uncommon, Magne et al.^32^ have advocated that a direct causality between this ratio and health status of the host is hard to be attested. A possible explanation for the detected differences in abundance of Firmicutes and Bacteroidetes is that bacteria positive to the Gram staining, like Firmicutes, are generally more vulnerable to the hydrolytic action of muramidases on PGN. Indeed, Firmicutes possess an undefended, thicker cell wall lacking an outer protecting lipid membrane and offering up to 40 PGN layers as a substrate to muramidases^8^. The lower Firmicutes abundance showed by MUH may explain the observed decrease in genes associated with peptidoglycan biosynthesis pathway. Moreover, the reduction in genes involved in amino sugar and nucleotide metabolism pathway, in which NAG is directly involved^33^, can be taken as another indicator for the inhibition of bacteria with high PGN synthesis capacity. MUH showed a considerable drop in Clostridiaceae. Interestingly, earlier studies on pigs^28^ and rabbits^12^ established that dietary supplementation of muramidases causes a depression in GI *Clostridia*. Furthermore, Sais et al.^23^ found a decreasing trend in *Clostridium* count at ileal level after supplementing broiler chickens with the muramidase tested here, yet at 35,000 LSU(F)/kg feed. MUH also exhibited a decrease in several butyrate-producing *Clostridia*, Lachnospiraceae (viz., *Roseburia intestinalis* and *Ruminococcus albus*), and *Eubacterium rectale*^34^. This was contrary to expectations as butyrogenic bacteria have traditionally been linked to gut health^35–37^, while chickens have been shown to benefit from short-chain fatty acids, especially butyrate, released by GI bacteria^38–41^. Future studies on this topic are therefore recommended. *C*. *perfringens* is the causative agent of necrotic enteritis, a gut disorder that causes to the poultry sector a financial burden of 2 billion dollars yearly^42^. Hence, the control of *C*. *perfringens* is vital, especially in the antibiotic growth promoters- and antimicrobial-free era. Even though the abundances found in this study were low, MUH showed a reduction in *C*. *perfringens*. This finding is consistent with that of Liu et al.^13^ who hindered the intestinal colonization of *C. perfringens* in broiler chickens orally challenged with this pathogen and treated with a dietary muramidase. Poultry are also susceptible to *C. botulinum* neurotoxins and can sporadically manifest avian botulism, a flaccid paralytic disease^43^. Despite the low abundances detected here, the decrease in *C. botulinum* exhibited by MUH is an issue that deserves further research. *L*. *monocytogenes*, an important human pathogen^44^, showed a minor presence in MUH although the measured abundances were rather low. Field studies revealed that poultry can be a reservoir of *L. monocytogenes*, thereby contributing to the contamination of processing facilities^45^. Interestingly, the observed inhibition of *C. botulinum* and *L. monocytogenes* supports earlier studies indicating that muramidases are effective solutions against these pathogenic bacteria^46,47^. The higher abundance of Bacteroidaceae in MUH agrees with results obtained in pigs^28,30^. However, contrary findings have also been found in the latter species^29^. *Bacteroides* have positively been associated to human gut health due to their propionate-producing ability^48,49^, while *B. thetaiotaomicron* has been included in a probiotic blend to restore gut eubiosis after antibiotic therapies^50^. Therefore, it can conceivably be posited that caecal Bacteroidaceae can promote intestinal health of broiler chickens as well. The observed increase in Lactobacillaceae is comparable to previous results on both non-avian species, like pigs^28–30^ and rabbits^12^, and broiler chickens supplemented with the muramidase used here^21,23^. The rise in Lactobacillaceae also differs from previous findings^10,19^ and can be contradictory considering the abovementioned proneness of Gram-positive bacteria to muramidase-mediated PGN hydrolysis. However, some lactic acid bacteria employed in the production of hard-cheese have been shown to be or gradually become resistant to muramidases^51,52^. Several Lactobacillaceae have also probiotic features^53^ that might have supported the performance of muramidase-supplemented birds, especially those treated at high dose. Attributing to this muramidase stimulating or, at least, non-inhibiting effects upon enteral Lactobacillaceae warrants further investigations.

Results of KEGG analysis can help interpret the changes occurred in the caecal metabolome. In MUH, the decrease in abundance of genes of starch and sucrose metabolism path can be behind the measured reduction in fermentations-deriving organic acids, such as acetate, ferulate, and formate. The enrichment in genes linked to glutathione metabolism path—a bacterial cells’ antioxidant tool^54^—can be associated with hypoxanthine drop at caecal level. Hypoxanthine is a noxious end-product of purine-catabolism, considered as a biomarker for oxidative stress^55–57^. Therefore, the lower concentration of hypoxanthine may have positively influenced the GI ecosystem of MUH birds. In addition, hypoxanthine decrease at intestinal level might have been the reason for its minor presence at plasma level. Lower circulating hypoxanthine, and histidine and uracil—protein- and nucleotide-catabolism end-products, respectively^56,57^—indicate that the degradation of proteins and nucleotides, intended to generate energy, may have occurred to a lesser extent in MUH birds. The higher abundance of caecal Bacteroidaceae may have increased the supply of propionate for the hepatic gluconeogenesis in MUH birds^58^, thereby leading to the observed rise in plasmatic energetic compounds such as pyruvate, 2-oxoglutarate, and glucose. The enrichment in bioenergetic compounds and reduction in prooxidant protein- and nucleotide-catabolites suggest that a more balanced energy metabolism may have stimulated the performance of high-dose supplemented birds. Surprisingly, cumulative FI, BW, and FCR were influenced in a dose-dependent fashion, while muramidase-supplemented groups showed the most marked microbiome and metabolome divergences. A possible explanation for this is that the cumulative performance benefited from an additive effect of each feeding phase, whereas the molecular outcomes at slaughter cannot fully justify the GI and metabolic dynamics of the entire grow-out period. Taken together, these findings contribute in several ways to our understanding of the mode of action of this dietary muramidase. The present study also lays the groundwork for future investigations on the effects of this muramidase on the GI ecosystem and systemic metabolism of broiler chickens.

## Methods

### Experimental design, housing, and husbandry conditions

A total of 2,340 day-old male Ross 308 broilers, obtained from the same breeder flock and hatching batch, were provided by a commercial hatchery. After hatch, they were vaccinated against infectious bronchitis virus, Marek’s disease virus, Newcastle and Gumboro diseases, and coccidiosis. Birds were housed in an experimental poultry facility, and randomly assigned to 3 groups (12 replicates/group) fed a commercial corn-wheat-soybean basal diet (control—CON) or the basal diet supplemented with a dietary muramidase (Balancius®, DSM Nutritional Products) at 25,000 (low-dose group—MUL) or 45,000 LSU(F)/kg feed (high-dose group—MUH) for the entire trial (0–42 d). Table 6 reports the basal diet formulation according to the 4-phase feeding program used (0–9 d, starter; 10–21 d, grower I; 22–28 d, grower II; 29–42 d, finisher). For each feeding phase, the mash basal diet was part of the same batch, while the powdery additive was added on top. The analytical inclusion levels of muramidase met the abovementioned targets. Each replicate was assigned to one of 36 floor pens (5.9 m^2^/pen) arranged in a randomized complete block design. Pens were equipped with two feeders, nipple drinkers, and chopped straw as bedding. Birds were manually fed and watered ad libitum on a daily basis. At each feeding phase switch—uniformly performed for all groups—feeders were emptied, cleaned, and refilled, while residuals weighed. The environmental temperature was modified according to the flock age by following the breeding company’s recommendations. The artificial photoperiod was 23L:1D during the first 7 and last 3 d, while 18L:6D for the remainder days. Birds were handled, raised, and processed in a commercial plant (42 d) in compliance with European Union legislation (Dir. 2007/43/EC; Reg. 2009/1099/EC; Dir. 2010/63/EU). The present study was approved by the Ethical Committee of the University of Bologna (ID: 1277).

**Table 6:** Basal diet composition according to feeding phases.

Ingredient (g/100 g)	Starter (0–9 d)	Grower I (10–21 d)	Grower II (22–28 d)	Finisher (29–42 d)
Corn	44.10	42.40	44.70	45.45
Wheat	10.10	15.00	15.00	15.00
Soybean meal	15.80	18.90	14.00	10.80
Pea	3.00	3.00	3.00	4.00
Fermented soybean meal	10.00	0.00	0.00	0.00
Full fat soybean	5.60	12.66	15.00	15.00
Sunflower meal	3.00	3.00	3.00	4.00
Corn gluten meal	3.00	0.00	0.00	0.00
Soybean oil	1.85	1.95	2.29	2.84
Calcium carbonate (39.5% Ca)	0.42	0.50	0.68	0.77
Dicalcium phosphate (25% Ca; 17% P)	1.07	0.63	0.37	0.22
Sodium bicarbonate (27% Na)	0.00	0.00	0.12	0.28
Sodium chloride (38% Na; 58.5% Cl)	0.33	0.31	0.24	0.17
Choline chloride	0.10	0.10	0.05	0.00
Lysine	0.52	0.47	0.41	0.38
Dl-methionine	0.22	0.13	0.22	0.10
MHA (methionine hydroxy analogue)	0.00	0.15	0.00	0.14
Threonine	0.14	0.15	0.13	0.11
NSP (non‐starch polysaccharides) enzyme	0.05	0.05	0.05	0.05
Phytase^†^	0.20	0.15	0.15	0.15
Natural pigments	0.00	0.00	0.24	0.24
Vitamin-mineral premix^‡^	0.50	0.45	0.35	0.30
**Composition (%)**^**§**^				
Dry matter	88.46	88.02	88.11	88.09
Protein	22.88	20.31	19.11	18.08
Lipid	4.99	6.37	7.24	7.81
Fibre	3.07	3.23	3.20	3.33
Ash	5.04	4.59	4.42	4.35
Lys (available)	1.26	1.15	1.05	0.97
Met + Cys (available)	0.91	0.84	0.78	0.74
Calcium (total)	0.72	0.61	0.59	0.58
Phosphorus (total)	0.57	0.49	0.43	0.40
**Energy content**^**§**^				
Metabolizable energy (kcal/kg)	3,020	3,097	3,172	3,222

^†^The premix included at 0.1% provides 1,000 FTU per kg of feed.

^‡^The premix provides the following per kg of feed: vitamin A (retinyl acetate), 13,000 IU; vitamin D3 (cholecalciferol), 4,000 IU; vitamin E (DL-α_tocopheryl acetate), 80 IU; vitamin K (menadione sodium bisulfite), 3 mg; riboflavin, 6.0 mg; pantothenic acid, 6.0 mg; niacin, 20 mg; pyridoxine, 2 mg; folic acid, 0.5 mg; biotin, 0.10 mg; thiamine, 2.5 mg; vitamin B_12_ 20 μg; Mn, 100 mg; Zn, 85 mg; Fe, 30 mg; Cu, 10 mg; I, 1.5 mg; Se, 0.2 mg; ethoxyquin, 100 mg.

^§^Calculated values.

### Performance traits measurement

On a replicate basis, the number and body weight (BW) of birds were recorded at housing (0 d), each feeding phase switch (10, 22, and 29 d), and slaughter (42 d), while feed intake (FI) for each feeding phase. Daily weight gain (DWG), daily feed intake (DFI), and feed conversion ratio (FCR) were calculated for each feeding phase and the whole rearing period (0–42 d). The number and BW of dead or culled birds were considered to correct performance data for mortality.

### Excreta collection and PGN hydrolysis assay

At the end of each feeding phase, fresh excreta samples were collected on a replicate basis (12 specimens/group; 36 specimens/feeding phase) and analyzed to evaluate bacterial PGN hydrolysis. The freeze-dried samples were resuspended and centrifuged. While the supernatant contained soluble PGN, the precipitate was enriched in insoluble PGN. Later, samples were subjected to acid hydrolysis to measure total and soluble PGN through liquid chromatography-mass spectrometric quantification of muramic acid used as a marker for hydrolyzed PGN (Novozymes A/S Biologiens, Lyngby, Denmark). Insoluble fraction of PGN was calculated as the difference between total and soluble PGN amount expressed as muramic acid. The tested muramidase has been shown to hydrolyze PGN of bacterial debris both in vitro and in ex vivo digesta samples of broiler chickens^25^. Therefore, the above described assay was performed to test, via a non-invasive method, the hypothesis of a larger proportion of hydrolyzed PGN in excreta of muramidase-treated birds.

### Processing traits, breast muscle myopathies, and foot-pad dermatitis evaluation

At slaughter (42 d) in a commercial plant, groups were clearly identified and separately processed. On a group basis, carcass and cut-up yields were measured on all processed birds according to standard commercial procedures. Occurrence of breast muscle myopathies, namely white striping (WS), wooden breast (WB), and spaghetti meat (SM), was evaluated—after chilling, deboning, and skin removal—on a randomly selected sample of approximately 150 breast fillets per group via a 3-point-scale: score 0, no abnormalities; score 1, moderate degree; score 2, severe degree^59^. Incidence of foot-pad dermatitis (FPD) was macroscopically measured on 1 foot per bird (i.e., more than 670 observations/group) by means of a 3-point scale: score 0, no lesions; score 1, mild lesions (≤ 0.8 cm); score 2, severe lesions (> 0.8 cm)^60^.

### Plasma and caecal content collection

From 1 bird per replicate (i.e., 12 birds/group), selected at slaughter (42 d) according to BW close to the flock average, blood and caecal content were sampled as previously described^61,62^. Briefly, blood was collected, poured into 4 mL lithium-heparin vials, and centrifuged (4,000 g; 900 s; 4 °C) to get plasma. Plasma samples were subsequently transferred into 1.5 mL tubes and stored at -80 °C until metabolomics analysis via proton nuclear magnetic resonance (^1^H-NMR). The content of both caeca was collected in duplicate into 1.5 mL sterile tubes that were then stored at -80 °C until ^1^H-NMR and DNA extraction for metagenome shotgun sequencing.

### DNA extraction, metagenome shotgun sequencing, and bioinformatics analysis

DNA extraction from caecal samples was performed via a bead-beating procedure using the QIAmp® DNA Stool Mini Kit (Qiagen, Milan, Italy) as previously illustrated^63^. Briefly, total extracted DNA was fragmented and tagged with sequencing indexes and adapters employing the Nextera XT DNA Library Preparation Kit (Illumina, San Diego, CA). Shotgun metagenomic sequencing was performed with NextSeq500 (Illumina) 2 × 150 bp in paired-end mode. Metagenomic sequencing yielded, on average, 6 Gbp per sample. Two out of 36 collected caecal content samples were excluded from subsequent bioinformatics analysis due to low sequencing yield. MG-RAST analysis server^64^ was utilized for the taxonomic identification by mapping sequencing reads against RefSeq database^65^. Moreover, Kyoto Encyclopedia of Genes and Genome (KEGG) database^66^ was selected for the hierarchical analysis of sequencing reads. Bacterial abundance matrix and KEGG matrix (down to species and KEGG level 3, respectively) were downloaded from MG-RAST and analyzed with R^67^ and STAMP v2.1.3^68^ as detailed below.

### Plasma and caecal content ^1^H-NMR analysis

An ^1^H-NMR analysis solution with D_2_O, containing 3-(trimethylsilyl)-propionic-2,2,3,3-d4 acid sodium salt (TSP) 10 mmol/L and NaN_3_ 2 mmol/L was made. The solution pH was set at 7.00 ± 0.02 by phosphate buffer 1 M. TSP was selected as a reference for NMR chemical-shift, while NaN_3_ was employed to avoid microorganism proliferation. Plasma samples were prepared for ^1^H-NMR analysis by centrifuging 1 mL of each sample (18,630 g; 900 s; 4 °C). 0.7 mL of supernatant was subsequently added to 0.1 mL of the ^1^H-NMR solution. Lastly, each sample was centrifuged once again as described above. Likewise, caecal content was prepared by vortex mixing approximately 80 mg of each sample with 1 mL of bi-distilled water: 0.7 mL of supernatant was treated as previously described for plasma.

^1^H-NMR spectra were registered (600.13 MHz; 298 K) with an AVANCE™ III spectrometer (Bruker, Milan, Italy), equipped with Topspin v3.5 software. We suppressed signals from broad resonances due to large molecules with a CPMG-filter composed by 400 echoes with a τ of 400 µs and a 180° pulse of 24 µs, for a total filter of 330 ms. The water residual signal was suppressed by means of presaturation. This was done by employing the *cpmgpr1d* sequence, part of the standard pulse sequence library. Each spectrum was acquired by summing up 256 transients constituted by 32,000 data points encompassing a window of 7184 Hz, separated by a relaxation delay of 5 s.

^1^H-NMR spectra were phase-adjusted in Topspin v3.5 and then exported to ASCII format by means of the built-in script *convbin2asc*. Spectra were processed with R software (R Core Team, 2020) through in-house developed scripts. We baseline-adjusted spectra by distinguishing baseline imperfection from NMR signals according to the “rolling ball” principle^69^ implemented in the R package *baseline*^70^.

Signal assignment was performed by comparing their chemical shift and multiplicity with Human Metabolome Database^71^ and Chenomx software library (Chenomx Inc., Edmonton, Canada, v10).

Plasma molecule concentrations were assessed by quantifying the molecules of the first sample analyzed by means of an external standard. Differences in water content between samples were then taken into consideration by probabilistic quotient normalization (PQN)^72^. Molecule concentrations in caecal samples were assessed as described for plasma by considering as reference the sample with the median water dilution assessed via PQN. The quantification of each molecule was performed through rectangular integration, considering one of its signals free from interferences.

### Statistical analysis

Performance data were analyzed through a one-way ANOVA with blocks, group as the experimental factor, and replicate as the experimental unit. Tukey’s honestly significant difference test was used to separate the groups’ means. Furthermore, polynomial contrasts were carried out to test for linear and quadratic trends in overall performance data (0–42 d). Data of carcass and cut-up yields were not subjected to statistical analysis because measured on a group basis. For each feeding phase, differences in soluble muramic acid, total muramic acid, and the ratio between them were analyzed through one-way ANOVA as described above. Pearson’s correlation coefficient between soluble to total muramic acid ratio and FCR data from each feeding phase was computed and tested for significance. Count data of FPD, WS, WB, and SM were analyzed by means of Pearson’s chi-square test and Fisher’s exact test involving all groups and using the sampled animal as experimental unit. All these analyses were performed in R^67^ with a significance level of 0.05. Moreover, FPD count data were arranged in 2 by 2 contingency tables aligning a combination of levels of the factor group (i.e., CON and MUL; CON and MUH; MUL and MUH) and having binarily aggregated FPD scores in columns (i.e., “FPD presence” as a sum of score 1 and score 2 counts; “FPD absence” as score 0 counts). Incidence risk ratio was computed on these 2 by 2 tables with *epiR*^73^ package of R^67^. If incidence risk ratio was significant at 95% confidence interval, the risk of developing FPD was computed as incidence risk ratio minus 1 and expressed as percentage. Ecological diversity indices were analyzed at genus level with *vegan*^74^ package of R^67^. Shannon, Simpson, and Inverse Simpson indices were chosen for alpha diversity, while the Bray–Curtis distance matrix method for beta diversity analysis. Alpha indices were analyzed with a one-way ANOVA and Tukey’s post-hoc test by considering the group as experimental factor and each sampled animal as experimental unit. Beta diversity was graphically explored through principal coordinates analysis (PCoA), and analyzed with PERMANOVA—“adonis” procedure with 10,000 permutations—followed by pairwise permutation MANOVA with *RVAideMemoire*^75^ package of R^67^. The matrix of caecal bacteria abundances was normalized for total read number in each sample and analyzed in STAMP v2.1.3^68^ by using Kruskal–Wallis H-test and Games-Howell post-hoc test with group as experimental factor and each sampled animal as experimental unit. The Firmicutes to Bacteroidetes ratio was calculated for each group and analyzed with one-way ANOVA and orthogonal contrasts (i.e., CON *vs* MUL + MUH and MUL *vs* MUH). KEGG level 3 matrix was filtered for “KEGG level 2”: this subset was then normalized and analyzed as described above. The significance level was set at 0.05. With respect to plasma and caecal metabolomics data, molecule concentrations were normalized via Box and Cox^76^ transformation. Then, a one-way ANOVA and Tukey’s post-hoc test were conducted considering the group as the experimental factor and each sampled animal as the experimental unit. To get an overview of metabolome trends, robust principal component analysis (rPCA)^77^ was performed on molecules showing significantly different between-group concentration in the abovementioned univariate analysis. Initially, PcaHubert algorithm—implemented in *rrcov* package of R^67^—detected outlying samples according to their distance from others along and orthogonally to the PCA plane. Later, the optimal number of PCs was determined. A score plot and a correlation plot summarized main features of the rPCA models. The former represents the samples in the PC space, thus evidencing the overall structure of data. The latter reports Pearson’s correlations between the concentration of each molecule and model components, thereby showing which molecule mostly affected the data structure. These analyses were carried out in R^67^ with a significance level of 0.05.

### Ethics declaration

All procedures involving animals were approved by the Ethical Committee of the University of Bologna (ID: 1277) and performed in accordance with European Union legislation (Dir. 2007/43/EC; Reg. 2009/1099/EC; Dir. 2010/63/EU) and ARRIVE guidelines.

## Data Availability

All data generated and analyzed during this study are included in this article and its additional files. Caecal microbiome sequences have been made public on MG-RAST repository with the project ID *Muramidase_UniBO_project_2020_34WGS* (https://www.mg-rast.org/linkin.cgi?project=mgp98274).

## Abbreviations

NAM: *N*-Acetylmuramic acid
NAG: *N*-Acetylglucosamine
PGN: Peptidoglycan
GI: Gastrointestinal
CON: Control group
MUL: Low-dose group
MUH: High-dose group
BW: Body weight
DWG: Daily weight gain
DFI: Daily feed intake
FI: Feed intake
FCR: Feed conversion ratio
FPD: Foot-pad dermatitis
WS: White striping
WB: Wooden breast
SM: Spaghetti meat
^1^H-NMR: Proton nuclear magnetic resonance
PCoA: Principal Coordinates Analysis
KEGG: Kyoto Encyclopedia of Genes and Genome
PQN: Probabilistic quotient normalization
rPCA: Robust principal component analysis

## References

[1] Gadde U, Kim WH, Oh ST, Lillehoj HS (2017). Alternatives to antibiotics for maximizing growth performance and feed efficiency in poultry: a review. Animal Health Res. Rev..

[2] Smits CHM, Li D, Patience JF, den Hartog LA (2021). Animal nutrition strategies and options to reduce the use of antimicrobials in animal production. FAO Animal Prod. Health Paper..

[3] Kiarie E, Romero LF, Nyachoti CM (2013). The role of added feed enzymes in promoting gut health in swine and poultry. Nutr. Res. Rev..

[4] Oviedo-Rondón EO (2019). Holistic view of intestinal health in poultry. Anim. Feed Sci. Technol..

[5] Wellman-Labadie O, Picman J, Hincke MT (2007). Avian antimicrobial proteins: Structure, distribution and activity. Worlds. Poult. Sci. J..

[6] Callewaert L, Michiels CW (2010). Lysozymes in the animal kingdom. J. Biosci..

[7] Nile CJ, Townes CL, Michailidis G, Hirst BH, Hall J (2004). Identification of chicken lysozyme g2 and its expression in the intestine. Cell. Mol. Life Sci..

[8] Masschalck B, Michiels CW (2003). Antimicrobial properties of lysozyme in relation to foodborne vegetative bacteria. Crit. Rev. Microbiol..

[9] May KD, Wells JE, Maxwell CV, Oliver WT (2012). Granulated lysozyme as an alternative to antibiotics improves growth performance and small intestinal morphology of 10-day-old pigs1. J. Anim. Sci..

[10] Ma, X., Zhang, S., Pan, L. & Piao, X. Effects of lysozyme on the growth performance, nutrient digestibility, intestinal barrier and microbiota of weaned pigs fed diets containing spray-dried whole egg or albumen powder. *Can. J. Anim. Sci.* **97**, CJAS-2016-0171 (2017).

[11] Xu S (2018). Effects of dietary supplementation with lysozyme during late gestation and lactation stage on the performance of sows and their offspring1. J. Anim. Sci..

[12] EL-Deep, M. H. *et al.* The influence of dietary chicken egg lysozyme on the growth performance, blood health, and resistance against *Escherichia coli* in the growing rabbits’ cecum. *Front. Vet. Sci.* **7**, 579576 (2020). 10.3389/fvets.2020.579576 PMC7593809 33195588

[13] Liu D, Guo Y, Wang Z, Yuan J (2010). Exogenous lysozyme influences Clostridium perfringens colonization and intestinal barrier function in broiler chickens. Avian Pathol..

[14] Gong M, Anderson D, Rathgeber B, MacIsaac J (2017). The effect of dietary lysozyme with EDTA on growth performance and intestinal microbiota of broiler chickens in each period of the growth cycle. J. Appl. Poult. Res..

[15] Xia, Y. *et al.* Effects of dietary supplementation with lysozyme on the structure and function of the cecal microbiota in broiler chickens. *PLoS ONE* **14**, e0216748 (2019). 10.1371/journal.pone.0216748 PMC6583987 31216277

[16] Ragland, S. A. & Criss, A. K. From bacterial killing to immune modulation: Recent insights into the functions of lysozyme. *PLOS Pathog.* **13**, e1006512 (2017). 10.1371/journal.ppat.1006512 PMC5608400 28934357

[17] Lee M (2009). Hen egg lysozyme attenuates inflammation and modulates local gene expression in a porcine model of dextran sodium sulfate (DSS)-induced colitis. J. Agric. Food Chem..

[18] Abdel-Latif, M. A. *et al.* Exogenous dietary lysozyme improves the growth performance and gut microbiota in broiler chickens targeting the antioxidant and non-specific immunity mRNA expression. *PLoS ONE* **12**, e0185153 (2017). 10.1371/journal.pone.0185153 PMC5653193 29059196

[19] Wang, Y. *et al.* Dietary muramidase degrades bacterial peptidoglycan to NOD-activating muramyl dipeptides and reduces duodenal inflammation in broiler chickens. *Br. J. Nutr.* 1–11 (2020) doi:10.1017/S0007114520004493. 10.1017/S0007114520004493 33172510

[20] Hall DM (2001). Mechanisms of circulatory and intestinal barrier dysfunction during whole body hyperthermia. Am. J. Physiol. Hear. Circ. Physiol..

[21] Lichtenberg J (2017). Safety evaluation of a novel muramidase for feed application. Regul. Toxicol. Pharmacol..

[22] Goodarzi Boroojeni, F., Männer, K., Rieger, J., Pérez Calvo, E. & Zentek, J. Evaluation of a microbial muramidase supplementation on growth performance, apparent ileal digestibility, and intestinal histology of broiler chickens. *Poult. Sci.* **98**, 2080–2086 (2019). 10.3382/ps/pey556 30566631

[23] Sais M (2020). Evaluation of dietary supplementation of a novel microbial muramidase on gastrointestinal functionality and growth performance in broiler chickens. Poult. Sci..

[24] Pirgozliev, V., Simic, A., Rose, S. P. & Pérez Calvo, E. Dietary microbial muramidase improves feed efficiency, energy and nutrient availability and welfare of broilers fed commercial type diets containing exogenous enzymes. *Br. Poult. Sci.* **62**, 131–137 (2021). 10.1080/00071668.2020.1817330 32875828

[25] Frederiksen CØ (2021). A muramidase from Acremonium alcalophilum hydrolyse peptidoglycan found in the gastrointestinal tract of broiler chickens. J. Ind. Microbiol. Biotechnol..

[26] Shepherd EM, Fairchild BD (2010). Footpad dermatitis in poultry. Poult. Sci..

[27] Sirri, F., Pignata, S., Franchini, A. & Meluzzi, A. Sweet chestnut tannin supplementation as a way to reduce the incidence of foot pad dermatitis of broiler chickens. in *Poster session presented at: 18th European Symposium on Poultry Nutrition; Oct 31–Nov 04* (2011).

[28] Maga EA (2012). Consumption of lysozyme-rich milk can alter microbial fecal populations. Appl. Environ. Microbiol..

[29] Xu S (2020). Fecal bacteria and metabolite responses to dietary lysozyme in a sow model from late gestation until lactation. Sci. Rep..

[30] Xiong X (2019). Dietary lysozyme supplementation contributes to enhanced intestinal functions and gut microflora of piglets. Food Funct..

[31] Wells JE (2015). Effect of lysozyme or antibiotics on faecal zoonotic pathogens in nursery pigs. J. Appl. Microbiol..

[32] Magne F (2020). The firmicutes/bacteroidetes ratio: a relevant marker of gut dysbiosis in obese patients?. Nutrients.

[33] Kyoto Encyclopedia of Genes and Genome. Compound C00140: N-Acetyl-D-glucosamine; N-Acetylchitosamine; 2-Acetamido-2-deoxy-D-glucose; GlcNAc. https://www.genome.jp/dbget-bin/www_bget?C00140.

[34] Rivière, A., Selak, M., Lantin, D., Leroy, F. & De Vuyst, L. Bifidobacteria and butyrate-producing colon bacteria: Importance and strategies for their stimulation in the human gut. *Front. Microbiol.* 7 (2016). 10.3389/fmicb.2016.00979 PMC4923077 27446020

[35] Koh A, De Vadder F, Kovatcheva-Datchary P, Bäckhed F (2016). From dietary fiber to host physiology: short-chain fatty acids as key bacterial metabolites. Cell.

[36] Milani C (2017). The first microbial colonizers of the human gut: composition, activities, and health implications of the infant gut microbiota. Microbiol. Mol. Biol. Rev..

[37] Vital, M., Karch, A. & Pieper, D. H. Colonic butyrate-producing communities in humans: an overview using omics data. *mSystems* **2**, e00130-17 (2017). 10.1128/mSystems.00130-17 PMC5715108 29238752

[38] Torok VA (2011). Identification and characterization of potential performance-related gut microbiotas in broiler chickens across various feeding trials. Appl. Environ. Microbiol..

[39] Stanley D (2012). Intestinal microbiota associated with differential feed conversion efficiency in chickens. Appl. Microbiol. Biotechnol..

[40] De Maesschalck C (2015). Effects of xylo-oligosaccharides on broiler chicken performance and microbiota. Appl. Environ. Microbiol..

[41] Stanley, D., Hughes, R. J., Geier, M. S. & Moore, R. J. Bacteria within the gastrointestinal tract microbiota correlated with improved growth and feed conversion: challenges presented for the identification of performance enhancing probiotic bacteria. *Front. Microbiol.* 7 (2016). 10.3389/fmicb.2016.00187 PMC4760072 26925052

[42] Van Immerseel F, Rood JI, Moore RJ, Titball RW (2009). Rethinking our understanding of the pathogenesis of necrotic enteritis in chickens. Trends Microbiol..

[43] Souillard R (2014). Investigation of Clostridium botulinum in commercial poultry farms in France between 2011 and 2013. Avian Pathol..

[44] European Food Safety Authority and European Centre for Disease Prevention and Control. The European Union One Health 2019 Zoonoses Report. *EFSA J.* **19** (2021). 10.2903/j.efsa.2021.6406 PMC7913300 33680134

[45] Rothrock MJ (2017). Listeria occurrence in poultry flocks: detection and potential implications. Front. Vet. Sci..

[46] Hughey VL, Johnson EA (1987). Antimicrobial activity of lysozyme against bacteria involved in food spoilage and food-borne disease. Appl. Environ. Microbiol..

[47] Hughey VL, Wilger PA, Johnson EA (1989). Antibacterial activity of hen egg white lysozyme against *Listeria monocytogenes* Scott A in foods. Appl. Environ. Microbiol..

[48] Rios-Covian, D., Salazar, N., Gueimonde, M. & de los Reyes-Gavilan, C. G. Shaping the metabolism of intestinal bacteroides population through diet to improve human health. *Front. Microbiol.* **8** (2017). 10.3389/fmicb.2017.00376 PMC5339271 28326076

[49] Jacobson A (2018). A gut commensal-produced metabolite mediates colonization resistance to salmonella infection. Cell Host Microbe.

[50] El Hage, R., Hernandez-Sanabria, E., Calatayud Arroyo, M., Props, R. & Van de Wiele, T. Propionate-producing consortium restores antibiotic-induced dysbiosis in a dynamic in vitro model of the human intestinal microbial ecosystem. *Front. Microbiol.* **10** (2019). 10.3389/fmicb.2019.01206 PMC6554338 31214145

[51] Bester BH, Lombard SH (1990). Influence of lysozyme on selected bacteria associated with gouda cheese. J. Food Prot..

[52] Neviani, E., Gatti, M., Tarelli, M. T. & Divizia, R. Lysozyme resistance of lactic acid bacteria. *Latte* 90–91 (1996).

[53] FAO. Probiotics in animal nutrition: production, impact and regulation. *FAO Anim. Prod. Heal. Pap. No. 179* (2016).

[54] Masip L, Veeravalli K, Georgiou G (2006). The many faces of glutathione in bacteria. Antioxid. Redox Signal..

[55] Beauclercq S (2016). Serum and muscle metabolomics for the prediction of ultimate pH, a key factor for chicken-meat quality. J. Proteome Res..

[56] Abasht, B., Mutryn, M. F., Michalek, R. D. & Lee, W. R. Oxidative stress and metabolic perturbations in wooden breast disorder in chickens. *PLoS ONE* **11**, e0153750 (2016). 10.1371/journal.pone.0153750 PMC4838225 27097013

[57] Soglia F, Silva AK, Lião LM, Laghi L, Petracci M (2019). Effect of broiler breast abnormality and freezing on meat quality and metabolites assessed by 1 H-NMR spectroscopy. Poult. Sci..

[58] Schwiertz A (2010). Microbiota and SCFA in lean and overweight healthy subjects. Obesity.

[59] Sirri F (2016). Effect of different levels of dietary zinc, manganese, and copper from organic or inorganic sources on performance, bacterial chondronecrosis, intramuscular collagen characteristics, and occurrence of meat quality defects of broiler chickens. Poult. Sci..

[60] Ekstrand, C., Carpenter, T. E., Andersson, I. & Algers, B. Prevalence and control of foot-pad dermatitis in broilers in Sweden. *Br. Poult. Sci.* 318–324 (1998) doi:10.1080/00071669888845. 10.1080/00071669888845 9693810

[61] Zampiga, M. *et al.* Effect of dietary arginine to lysine ratios on productive performance, meat quality, plasma and muscle metabolomics profile in fast-growing broiler chickens. *J. Anim. Sci. Biotechnol.* **9**, (2018). 10.1186/s40104-018-0294-5 PMC6223088 30455879

[62] Brugaletta G (2020). Insights into the mode of action of tannin-based feed additives in broiler chickens: looking for connections with the plasma metabolome and caecal microbiota. Ital. J. Anim. Sci..

[63] De Cesare A (2017). Effect of dietary supplementation with Lactobacillus acidophilus D2/CSL (CECT 4529) on caecum microbioma and productive performance in broiler chickens. PLoS ONE.

[64] Meyer F (2008). The metagenomics RAST server: a public resource for the automatic phylogenetic and functional analysis of metagenomes. BMC Bioinform..

[65] O’Leary NA (2016). Reference sequence (RefSeq) database at NCBI: current status, taxonomic expansion, and functional annotation. Nucl. Acids Res..

[66] Kanehisa M, Goto S (2000). KEGG: kyoto encyclopedia of genes and genomes. Nucl. Acids Res..

[67] R Core Team. R: A language and environment for statistical computing. (2020).

[68] Parks DH, Tyson GW, Hugenholtz P, Beiko RG (2014). STAMP: Statistical analysis of taxonomic and functional profiles. Bioinformatics.

[69] Kneen, M. A. & Annegarn, H. J. Algorithm for fitting XRF, SEM and PIXE X-ray spectra backgrounds. *Nucl. Instruments Methods Phys. Res. Sect. B Beam Interact. Mater. Atoms* **109**–**110**, 209–213 (1996).

[70] Liland KH, Almøy T, Mevik BH (2010). Optimal choice of baseline correction for multivariate calibration of spectra. Appl. Spectrosc..

[71] Wishart, D. S. *et al.* HMDB: The human metabolome database. *Nucl. Acids Res.* **35**, (2007). 10.1093/nar/gkl923 PMC1899095 17202168

[72] Dieterle, F., Ross, A., Schlotterbeck, G. & Senn, H. Probabilistic quotient normalization as robust method to account for dilution of complex biological mixtures. Application in 1 H NMR metabonomics. *Anal. Chem.* **78**, 4281–4290 (2006). 10.1021/ac051632c 16808434

[73] Stevenson, M. *et al.* epiR: tools for the analysis of epidemiological data. R package version 2.0.19. (2021).

[74] Oksanen, J. *et al.* vegan: community ecology package. R package version 2.5–6. (2020).

[75] Hervé, M. RVAideMemoire: testing and plotting procedures for biostatistics. (2021).

[76] Box GEP, Cox DR (1964). An analysis of transformations. J. R. Stat. Soc. Ser. B.

[77] Hubert, M., Rousseeuw, P. J. & Vanden Branden, K. ROBPCA: a new approach to robust principal component analysis. *Technometrics* **47**, 64–79 (2005).

